# Effects of maternal influenza vaccination on adverse birth outcomes: A systematic review and Bayesian meta-analysis

**DOI:** 10.1371/journal.pone.0220910

**Published:** 2019-08-14

**Authors:** Sohyun Jeong, Eun Jin Jang, Junwoo Jo, Sunmee Jang

**Affiliations:** 1 Hinda and Arthur Marcus Institute for Aging Research, Hebrew SeniorLife and Harvard Medical School, Boston, Massachusetts, United States of America; 2 School of Pharmacy, Sungkyunkwan University, Jangan-gu, Suwon, Gyeonggi-do, Korea; 3 Department of Information Statistics, Andong National University, Gyeongsangbuk-do, Korea; 4 Department of Statistics, Kyungpook National University, Bukgu, Daegu, Korea; 5 College of Pharmacy and Gachon Institute of Pharmaceutical Sciences, Gachon University, Incheon, Korea; Universita degli Studi di Ferrara, ITALY

## Abstract

**Background:**

Although pregnant women are a priority group for influenza vaccination, its effect on birth outcomes has long been debated. Numerous observational studies and a few randomized controlled studies have been conducted, with inconsistent results.

**Objectives:**

To evaluate the association of influenza vaccination in pregnancy with adverse birth outcomes.

**Data source:**

The Cochrane Library, PubMed, EMBASE, Web of Science, and Scopus were searched.

**Study eligibility criteria:**

This analysis included randomized placebo-controlled studies, cohort studies, and case-control studies, in which inactivated influenza vaccination was given during pregnancy and fetal adverse birth outcomes were assessed.

**Participants & intervention:**

Women who received inactivated influenza vaccine during pregnancy and their offspring.

**Study appraisal and synthesis:**

Two independent reviewers and a third reviewer collaborated in study selection and data extraction. A Bayesian 3-level random-effects model was utilized to assess the impact of maternal influenza vaccination on birth outcomes, which were presented as odds ratios (ORs) with 95% credible interval (CrIs). Bayesian outcome probabilities (P) of an OR<1 were calculated, and values of at least 90% (0.9) were deemed to indicate a significant result.

**Results:**

Among the 6,249 identified publications, 48 studies were eligible for the meta-analysis, including 2 randomized controlled trials, 41 cohort studies, and 5 case-control studies. The risk of none of the following adverse birth outcomes decreased significantly: preterm birth (OR = 0.945, 95% CrI: 0.736–1.345, P = 73.3%), low birth weight (OR = 0.928, 95% CrI: 0.432–2.112, P = 76.7%), small for gestational age (OR = 0.971, 95% CrI: 0.249–4.217,P = 63.3%), congenital malformation (OR = 1.026, 95% CrI: 0.687–1.600, P = 38.0%), and fetal death (OR = 0.942, 95% CrI: 0.560–1.954, P = 61.6%). Summary estimates including only cohort studies showed significantly decreased risks for preterm birth, small for gestational age and fetal death. However, after adjusting for season at the time of vaccination and countries’ income level, only fetal death remained significant.

**Conclusion:**

This Bayesian meta-analysis did not find a protective effect of maternal influenza vaccination against adverse birth outcomes, as reported in previous studies. In fact, our results showed evidence of null associations between maternal influenza vaccination and adverse birth outcomes.

## Introduction

Pregnant women are deemed vulnerable to severe complications from influenza infection due to changes in the immune system and heart and lung function during pregnancy [1]. Influenza infection can also affect fetal development, leading to adverse birth outcomes [2, 3]. Based on the evidence of the landmark Mother’s Gift trial in Bangladesh [4], the World Health Organization (WHO) Strategic Advisory Group for Experts on Immunization recommended in 2012 that pregnant women be considered a priority group for influenza vaccination [5]. Many high-income countries have adopted and incorporated the WHO recommendations in their maternal-child health strategies [6]. Numerous studies have shown inactivated influenza vaccination in pregnant women to be effective at protecting women and their offspring via transplacental antibody transfer [7, 8]. The two-fold benefit of inactivated influenza vaccine in preventing laboratory-confirmed influenza infection, both in pregnant women and infants, is convincing [9, 10], especially as it has been proven in recent, large-scale, phase 4 randomized controlled trials (RCTs) conducted in Mali [11], Nepal [12], and South Africa [10, 13].

However, the influenza vaccine uptake rate varies widely, ranging between 4% and 93% [14, 15], and concerns about the risk to the fetus are a major reason why women sometimes do not receive vaccination during pregnancy, especially in many low/middle-income countries where maternal influenza vaccination has not been incorporated into routine immunization programs [16, 17]. To present solutions for this issue, many phase 4, post-marketing surveillance or observational studies have been conducted to evaluate the fetal outcomes of preterm birth (PTB), small for gestational age (SGA), low birth weight (LBW), congenital abnormalities and fetal death. Many of those studies have suggested that influenza vaccination during pregnancy was not associated with adverse birth outcomes [18–36], but some reported negative [37] or positive [38–46] results for each of the above birth outcomes. A few systematic reviews and meta-analyses were conducted, with similarly inconsistent results; maternal influenza vaccination reduced the incidence of PTB [39, 47], SGA, LBW [47], and stillbirth (or fetal death) [39, 48], but no association was found with congenital malformations [49, 50], fetal death, or spontaneous abortion [49]. However, Vazquez-Benitez et al. raised the issue that biases in conducting observational studies of maternal influenza vaccination may lead to false positives [51]. Donzelli [52] also explicitly stated that positive results from observational studies might be due to confounding by indication and healthy-vaccine bias. Furthermore, a case-control study funded by the US Centers for Disease Control and Prevention (CDC) reported that women who were given maternal flu vaccination containing the pandemic H1N1 (H1N1pdm09) component in the prior season had an increased risk of spontaneous abortion within 28 days after the next seasonal vaccination [53]. Research is continuing into this issue, and the CDC has not confirmed that the maternal flu vaccine causes spontaneous abortion [54].

These results indicate that the evidence for adverse birth outcomes is inconsistent and lacking methodological robustness, as it is largely dependent on observational studies. Critiques regarding the limitations of ecological studies—including statistical imprecision and clinical and methodological heterogeneity—that make them prone to substantial bias have argued that more sound research is needed, in addition to previously published systematic reviews and meta-analyses [55]. Therefore, given the large volume of observational studies and the paucity of RCTs available, we aimed to overcome the heterogeneity of studies by conducting a Bayesian hierarchical meta-analysis, and to present new evidence regarding the association between maternal influenza vaccination and adverse fetal birth outcomes.

## Methods

### Search strategy

A systematic literature review was conducted conforming to the Preferred Reporting Items for Systematic Reviews and Meta-Analyses (PRISMA) recommendations [56]. The Cochrane Library, PubMed, EMBASE, Web of Science, and Scopus were searched using the following keywords: “pregnan*”, “influenza”, “vaccine,” and “immunization,” filtered with ‘human’ without limitations on the date up to May 2019. A manual review was performed to search for other relevant articles in the references of selected articles and WHO publications. The inclusion criteria were 1) human studies; 2) inactivated influenza vaccination given in any period of pregnancy; 3) reporting the target outcomes of PTB, LBW, SGA, fetal death, and congenital malformations; 4) RCTs, prospective or retrospective cohort studies, case-control studies; 5) placebo-controlled studies, and 6) the availability of full-text published articles in the English language. The exclusion criteria were 1) single-arm studies; 2) cross-sectional studies; 3) active controlled studies; 4) animal studies; 5) studies not on the target outcome, target vaccine, or target population; 6) reviews, abstracts for posters, editorials, commentaries, or letters; 7) studies not written in English; and 8) studies with no available full text.

Two independent reviewers (MS & JL) searched for and selected the final studies through consensus. An additional third reviewer (SJ) also participated in the process and reached the same consensus on the final selection.

### Data items

We retrieved relevant information from the final selected studies: publication year, name of the first author, total number of participants, vaccine type (trivalent, monovalent, or MF59-adjuvant), the period when vaccines were given (pandemic or seasonal), country of the study, income level of the country where the study was conducted, study design, and the effect sizes and confidence intervals reported in the studies.

### Assessment of risk of bias and publication bias

The quality of the included studies was independently assessed by 2 researchers (JL & MS). RCTs were graded by the Risk of Bias tool developed by the Cochrane Library, whereas cohort and case-control studies were assessed by the Newcastle-Ottawa Scale (NOS). Funnel plot visualization and the Egger regression method were used, since they are widely accepted tools for assessing publication bias.

### Statistical analysis: Bayesian meta-analysis

A Bayesian meta-analysis was performed to assess the association between adverse birth outcomes and influenza vaccination during pregnancy. The Bayesian approach is superior to traditional meta-analyses, especially for rare adverse events [57]. In the Bayesian method, the posterior probability distribution is obtained by combining a prior distribution with a likelihood distribution from the observed data, and the quantity of interest can be directly interpreted as the probability [58]. Since this study included cohort studies, case-control studies, and RCTs, to consider the heterogeneity among study design, a hierarchical Bayesian 3-level random-effects model was used [59]. A hierarchical Bayesian random-effects model is useful when incorporating the imprecision of the variance estimates of treatment effects within studies, the imprecision in the estimated between-study variance estimate, and explicitly modeling binary outcome data [60]. The odds ratios (ORs) and 95% confidence intervals (CIs) or sample size and the count of the outcome were extracted from each study. When an adjusted OR was presented in the cohort study or case-control study, it was first extracted and used as the effect size. When the effect size was presented as a hazard ratio (HR) or relative risk (RR), it was considered as an OR. Since the underlying assumption of a generally low event risk (<20%) can be made for adverse birth events, and the HRs and RRs are very close to 1, it can be logically concluded that HRs, RRs, and ORs will be relatively close to each other [61–63]. ORs were calculated when the sample size and the number of outcomes were present in a study, and ORs were calculated after a continuity correction when there were no adverse birth outcomes in the vaccinated pregnant women or the unvaccinated pregnant women. The ORs calculated for each study were converted to log (OR) values, which were used as the effect size in the Bayesian meta-analysis.

We presented the posterior median and 95% credible interval (CrI) of the overall ORs and ORs for each study design (RCTs, cohort studies, case-control studies). The main goal of this study was to investigate whether the risk of targeted birth outcomes was lower in the vaccinated group than in the unvaccinated group. To verify this hypothesis, the posterior probability (P) of the OR being less than 1, which can also be denoted as P(OR<1), was estimated. Since a probability of 50% can be interpreted as a null effect, whereas a probability of at least 90% indicates a significantly beneficial effect of maternal vaccination in decreasing adverse birth outcomes [64], a probability of over 90% was considered significant in this study. To explore the effects of covariates such as the vaccination period (seasonal or pandemic) and the income level of the country where the study was conducted, Bayesian meta-regression was performed. If the 95% CrI of regression coefficient did not contain 0, the covariates were deemed statistically significant.

The non-informative prior distribution was considered as the prior distribution, and the convergence of the Markov-chain Monte Carlo (MCMC) algorithm was assessed using the trace plot, autocorrelation plot, and the Gelman-Rubin statistic[65]. In total, 110,000 iterations were performed in each of three chains. The first 10,000 iterations were burn-in to eliminate the initial value effect, and each 10th value among the remaining 100,000 values was used to estimate the posterior distribution. When the posterior median of the standard deviation among studies (heterogeneity, τ) was equal to or lower than 1, the heterogeneity was deemed non-significant [66, 67]. The funnel plot method and the Egger test were used to explore publication bias in the included studies. All models were fitted in WinBUGs 1.4.3 (Medical Research Council Biostatistics Unit, Cambridge, UK) using MCMC algorithms.

## Results

### PRISMA flow for study selection

**Fig 1:**
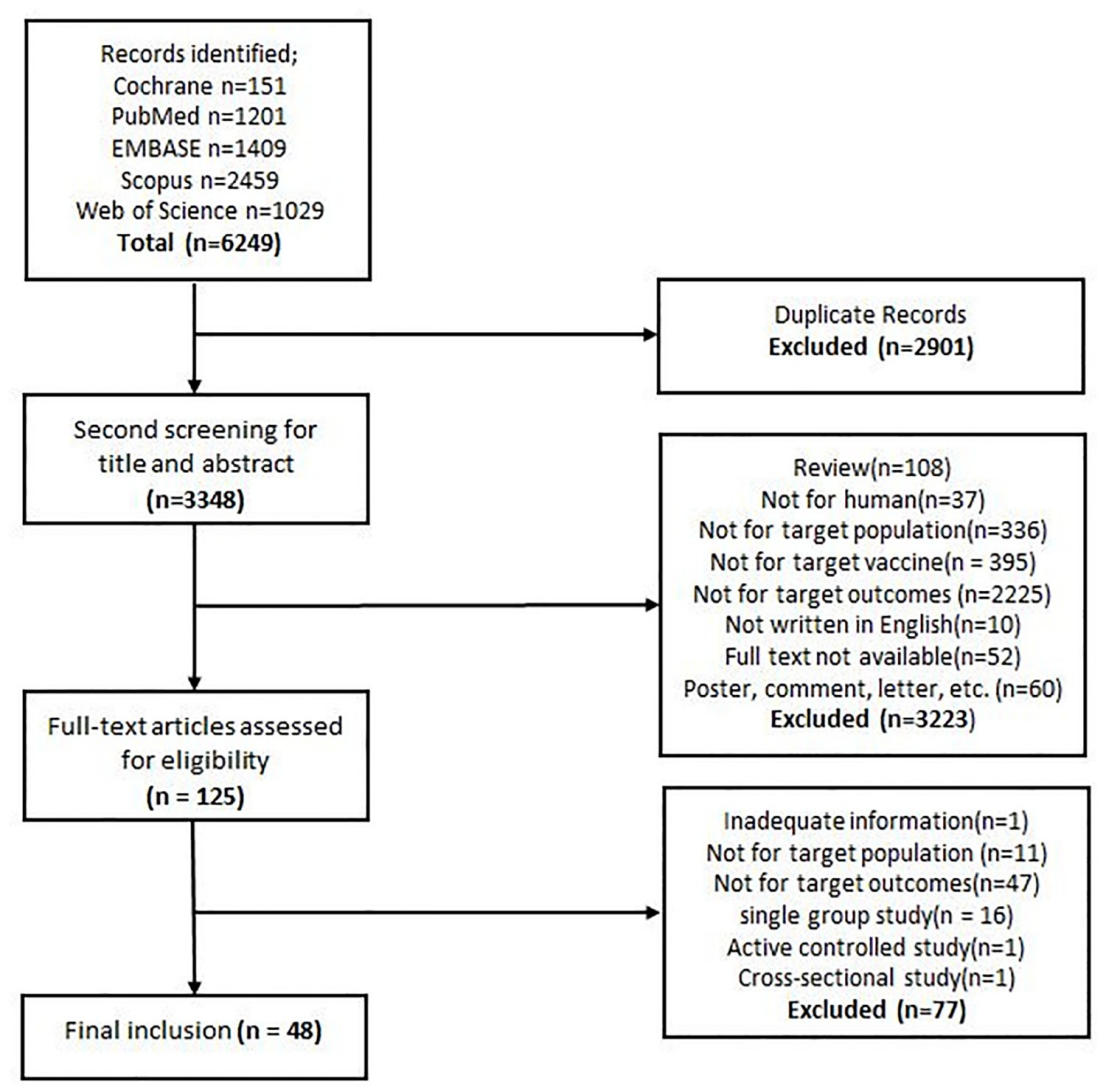
PRISMA flow diagram of study selection for the meta-analysis.

Among the 6,249 identified publications, 48 studies were eligible for the meta-analysis. Duplicate articles (n = 2,901) were excluded in the first step, and 3,223 articles were removed as inappropriate by title and abstract review. One hundred and twenty-five articles were eligible for full-text review. After excluding studies that did not investigate our target population (pregnant women or their infants) (n = 11) or the targeted birth outcomes (n = 47); provided inadequate information (n = 1); or were single-group studies (n = 16), an active controlled study (n = 1), or a cross-sectional study (n = 1), 48 studies were finally selected for the meta-analysis (Fig 1).

### Characteristics of included studies

Two RCTs, 5 case-control studies, and 41 cohort studies were included. According to the world income data of the period when the studies were conducted, 6 of them were from lower/middle-income countries [68] including 2 large RCTs (Nepal, South Africa), China, Nicaragua, Taiwan and Laos. Forty-one studies were from high-income countries [68], mainly located in North America and Western Europe (S1 Table).

Twenty-five studies reported birth outcomes associated with monovalent pandemic vaccination, 10 studies reported outcomes from the trivalent inactivated influenza vaccine, and the rest either did not indicate the vaccine type or indicated both types. A total of 2,387,107 pregnant women or their offspring were included in the 48 studies. The smallest study had 226 subjects and the largest had 425,944 subjects. The source of observational studies was generally pregnancy or birth or prenatal registries, vaccine registries, vaccine safety data linkage, hospital records, and surveillance data.

### Quality assessment of included studies: Risk of bias and publication bias

The quality of the included studies was independently assessed by 2 researchers. RCTs were graded by the Risk of Bias tool developed by the Cochrane Library [69] (S1 Fig), whereas the NOS [70] was used to check the quality score of cohort studies and case-control studies. As the selected primary literature had a low risk of bias in the domain of baseline outcome measures and characteristics, the baseline characteristics between the two groups were similar. The reporting of results likewise had little risk. However, the risks regarding blinding, allocation concealment, and contamination were high due to the nature of observational studies. Additionally, we stratified the NOS quality score of the included observational studies according to the Agency for Healthcare Research and Quality (AHRQ) conversion standards [71]. Among 46 observational studies, 37 were of good quality and 9 studies were of fair quality (S2 Table).

As widely accepted tools for publication bias, funnel plot visualization and the Egger regression method were used. The Egger regression method did not detect publication bias for PTB (*p*:*0*.*709*), LBW (*p*:*0*.*309*), SGA(*p*:*0*.*203*), congenital malformations(*p*:*0*.*972*) or fetal death (*p*:*0*.*670*). Funnel plots of all the analyses suggested that most studies had a large sample size, clustering in the upper part of the funnel plot. The presence of no studies in the lower-left part of the funnel plot for LBW suggests that small studies finding an increased risk of LBW may have remained unpublished (S2 Fig).

### Association between maternal influenza vaccination and adverse birth outcomes

Each birth outcome was assessed by the Bayesian random-effects model and the risk of bias across studies (heterogeneity) was assessed by the posterior median of standard deviation among studies (SD, τ). All the studies included and evaluated in each adverse outcome showed low heterogeneity (SD close to 0) (Table 1). The effects of the vaccination period (seasonal or pandemic) and the income level of the country where the study was conducted on each birth outcome were evaluated by meta-regression analyses. Only covariate of high income country in PTB analysis (beta: -0.141, 95% CrI: -0.276-(-0.004)) showed significantly protective effect and none of the other covariates showed statistically significant effects, as the 95% CrIs for their coefficients (β) included 0 (S3 Table). However, after adjusting for the effects of covariates in cohort summary estimates, the probability (P) of protection against PTB, LBW and SGA was significantly reduced and only protective effect against fetal death remained significant (Table 1).

**Table 1:** Summary results of Bayesian random-effect and meta-regression models on birth outcomes affected by maternal influenza vaccination.

Birth outcomes	Bayesian random-effect model					Bayesian random-effect meta-regression model							
	Study	Odds ratio			P (OR<1)	Odds ratio (vaccination season)			P (OR<1)	Odds ratio (income level)			P (OR<1)
		Posterior median	95% CrI			Posterior median	95% CrI			Posterior median	95% CrI		
PTB	SD (τ)	0.094				0.101				0.086			
	Overall	0.945	0.736	1.345	0.733	0.837	0.566	1.278	0.844	1.019	0.784	1.364	0.414
	RCT overall	0.954	0.787	1.224	0.707	0.847	0.606	1.201	0.848	1.003	0.816	1.260	0.484
	Cohort overall	0.916	0.859	0.977	0.995	0.798	0.608	1.029	0.960	1.016	0.905	1.143	0.393
	Case-control overall	0.970	0.762	1.439	0.601	0.868	0.593	1.369	0.765	1.039	0.804	1.449	0.359
LBW	SD (τ)	0.107				0.119				0.120			
	Overall	0.928	0.432	2.112	0.766	0.508	0.045	4.655	0.714	0.952	0.405	2.335	0.642
	RCT overall	0.926	0.721	1.238	0.800	0.500	0.066	3.669	0.727	0.938	0.714	1.301	0.723
	Cohort overall	0.932	0.864	0.998	0.978	0.521	0.072	3.736	0.715	0.966	0.786	1.165	0.644
SGA	SD (τ)	0.123				0.124				0.125			
	Overall	0.971	0.249	4.217	0.633	1.202	0.273	4.783	0.728	0.975	0.205	4.482	0.608
	RCT overall	0.964	0.475	2.010	0.666	1.189	0.538	2.685	0.264	0.965	0.478	2.051	0.653
	Cohort overall	0.976	0.947	1.004	0.955	1.211	0.798	1.892	0.851	0.981	0.927	1.039	0.768
Con-genital mal-formation	SD (τ)	0.100				0.103				0.102			
	Overall	1.026	0.687	1.600	0.380	1.383	0.814	2.459	0.086	1.005	0.643	1.577	0.480
	RCT overall	1.032	0.659	1.709	0.390	1.393	0.795	2.576	0.095	1.002	0.610	1.621	0.494
	Cohort overall	1.026	0.955	1.108	0.220	1.384	0.961	2.058	0.040	1.007	0.891	1.173	0.451
	Case-control overall	1.022	0.706	1.499	0.410	1.374	0.827	2.347	0.087	1.004	0.677	1.504	0.487
Fetal death	SD (τ)	0.222				0.191				0.203			
	Overall	0.942	0.560	1.954	0.616	0.732	0.372	1.681	0.821	0.977	0.578	1.885	0.550
	RCT overall	1.002	0.686	1.679	0.497	0.767	0.414	1.525	0.791	1.023	0.693	1.673	0.454
	Cohort overall	0.822	0.718	0.931	0.996	0.643	0.391	1.100	0.951	0.857	0.655	1.102	0.919
	Case-control overall	1.077	0.719	1.833	0.385	0.814	0.433	1.672	0.726	1.098	0.724	1.832	0.343

CrI: Credible Interval,

SD (τ): Standard deviation between studies (estimation of heterogeneity between studies)

#### Bayesian meta-analysis of PTB

**Fig 2:**
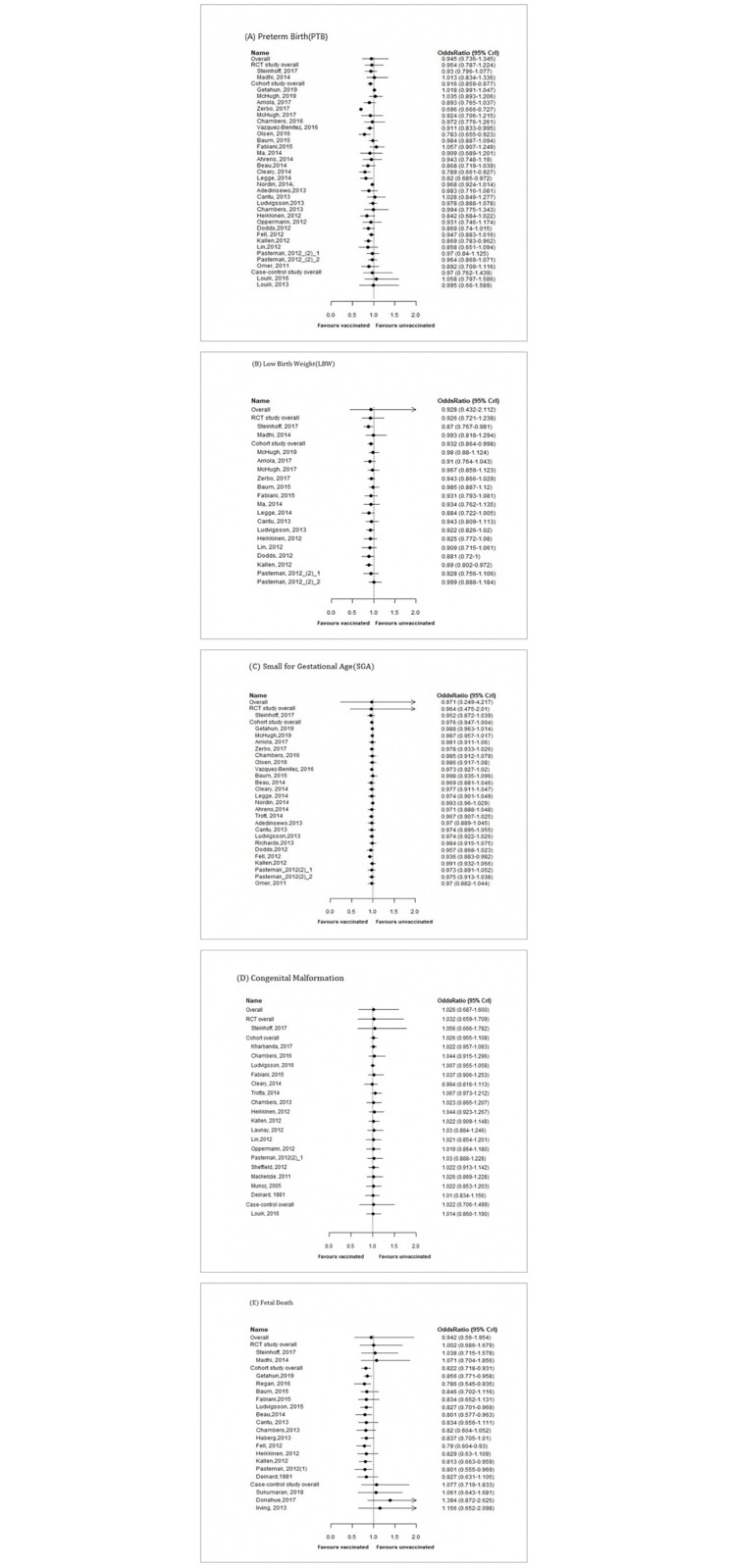
The results of meta-analyses of the associations between maternal influenza vaccination and adverse birth outcomes: (A) preterm birth, (B) low birth weight, (C) small for gestational age, (D) congenital malformation, (E) fetal death.

Thirty-two studies, 2 RCTs, 28 cohort studies (1 cohort study [18] reported results from 2 different groups, so 29 data sets were included), and 2 case-control studies were included in the PTB analysis (τ = 0.094,low heterogeneity). The overall PTB risk was not significantly decreased in the vaccinated group (OR = 0.945, 95% CrI: 0.736–1.345, P = 73.3%). The risk of PTB in RCTs and case-control studies was not significantly decreased in the vaccinated group. The PTB risk in cohort studies was significantly lower in the vaccinated group (OR = 0.916, 95% CrI: 0.859–0.977, P = 99.5%) but adjusting for income level of the country in cohort estimates made the association insignificant (Table 1, Fig 2(A), S4(A) Table).

#### Bayesian meta-analysis of LBW

Seventeen studies, including 2 RCTs and 15 cohort studies (16 data sets were included from 15 cohort studies, as mentioned in the above section) were included in the LBW analysis (τ = 0.107, low heterogeneity). The overall results did not satisfy the criterion of a P(OR<1) over 90% (OR = 0.928, 95% CrI: 0.432–2.112, P = 76.6%). The results from RCTs likewise did not show a decreased LBW risk in the vaccinated group. The risk of LBW in cohort studies was significantly lower in the vaccinated group (OR = 0.932, 95% CrI: 0.864–0.998, P = 97.8%). However, adjusting for covariates by meta-regression nullified the significance in the analyses of both covariates (Table 1, Fig 2(B), S4(B) Table).

#### Bayesian meta-analysis of SGA

One RCT and 23 cohort studies (24 data sets were included from 22 cohort studies as mentioned in the above section) were included in the SGA analysis (τ = 0.123, low heterogeneity). The overall results for the risk of SGA were not significant (OR = 0.971, 95% CrI: 0.249–4.217, P = 63.3%). The summary results from RCTs did not show significance. However, the cohort studies showed a significantly lower risk of SGA (OR = 0.976, 95% CrI: 0.947–1.004, P = 95.5%) in the vaccinated group than in the unvaccinated group. However, after adjusting for each covariate, the probabilities of a decreased risk of SGA were considerably reduced and became insignificant (Table 1, Fig 2(C), S4(C) Table).

#### Bayesian meta-analysis of congenital malformations

Nineteen studies, comprising 1 RCT, 17 cohort studies, and 1 case-control study, were included in the congenital malformation analysis (τ = 0.100, low heterogeneity). The overall summary results of congenital malformation presented a trend for higher risk in the vaccinated group than in the unvaccinated group, but without significance (OR = 1.026, 95% CrI: 0.687–1.600, P = 38.0%). None of the summary results from RCTs, cohort studies, and case-control studies were significant. No covariate (season or income level) significantly affected the association (Table 1, Fig 2(D), S4(D) Table). Since the information on the gestational period when the vaccination was given was only available for congenital malformation, it was analyzed as covariate for this birth outcome. Gestational period also did not have any influence on the risk of congenital malformation. The overall summary effect after adjusting for gestation periods was as follows; OR: 0.865, 95% CrI: 0.192–2.757, P = 61.8% (S5 Table).

#### Bayesian meta-analysis of fetal death

Nineteen studies were included in the analysis of fetal death, including 2 RCTs, 14 cohort studies, and 3 case-control studies (τ = 0.165, low heterogeneity). The overall risk of fetal death was not significantly decreased in the vaccinated group (OR = 0.942, 95% CrI: 0.560–1.954, P = 61.6%). The summary results from RCTs and case-control studies did not show a significant association. However, cohort studies showed a significant reduction of the fetal death risk (OR = 0.822, 95% CrI: 0.718–0.931, P = 99.6%). Meta-regression on covariates did not modify the final association (Table 1, Fig 2(E), S4(E) Table).

## Discussion

In this study, we found that inactivated influenza vaccination during pregnancy was neither associated with nor protective against the adverse birth outcomes of PTB, LBW, SGA, fetal death, and congenital malformations. We summarized results from 48 studies analyzing over 2 million subjects (pregnant women and infants). Overall, no association was found, except for the summary estimates of only cohort studies, which suggested protective effects against PTB (after adjusting for season at the time of vaccination) and fetal death(after adjusting for each covariate; season at the time of vaccination and countries’ income level). The most notable finding of this study is that we did not replicate previous findings of the beneficial effects of maternal influenza vaccination on PTB, LBW, and fetal death, which a few previous systematic reviews and meta-analyses have reported [39, 47, 48]. Additionally, we found that maternal influenza vaccination was not associated with the incidence of any adverse birth outcomes, which goes hand in hand with ensuring the safety of influenza vaccination during pregnancy. The safety of maternal vaccination has always been a sensitive issue, and a report of a two-fold increased risk of spontaneous abortion (SAB) in vaccinated women in a case-control study conducted by Donahue et al. has recently received attention [72]. That study triggered renewed arguments about the safety of maternal influenza vaccination. Donahue et al. reported an increased risk of spontaneous abortion (adjusted OR = 2.0; 95% CI, 1.1–3.6) in women who received an influenza vaccine 1–28 days before the diagnosis of spontaneous abortion. In response to the subsequent criticism and debates about their report, the authors defended their findings, stating that despite not being able to escape from the limitation of residual confounding, the considerable robustness of their study design implied that the increased SAB risk found in their study was not a false positive[73]. Although we could not evaluate the risk of fetal death in the period examined by Donahue et al. due to limitations in the available information, we still analyzed the most recent high-quality publications, and on that basis might suggest that the inactivated influenza vaccine administered during pregnancy did not increase the risk of fetal death.

Two randomized placebo-controlled studies investigated the efficacy and safety of maternal influenza vaccination in South Africa and South Asia, respectively. Madhi et al. (2014) [10] reported higher rates of stillbirth (1.4% vs. 0.9%), PTB (10.5% vs. 9.4%), and LBW (13% vs. 12%) in the vaccinated group than in the unvaccinated group, but did not present statistical significance. Steinhoff et al. (2017) [12] reported that maternal influenza vaccination reduced the LBW rate by 15% (95% CI, 3%–25%) but did not significantly affect the rate of SGA. They also reported low proportions of stillbirth (1.7% vs. 1.8%) and congenital defects (1.0% vs. 1.1%), but did not specify the significance.

The meta-analysis of Nunes et al. (2016), comprising 18 observational studies [47], reported that inactivated influenza vaccination during pregnancy had protective effects on PTB and LBW. Inactivated influenza vaccine reduced the risk of PTB and LBW by about 8%-13% and 12%-26%, respectively [47]. Another systematic review by Fell et al. (2014) included 1 RCT and 26 observational studies. They reported a modestly reduced risk of fetal death and PTB, noting the need for a cautious interpretation of the protective effect due to the shortcomings of observational studies [39]. Bratton et al. (2014) assessed 7 observational articles on fetal death (stillbirth or spontaneous abortion). They concluded that vaccinated women had about a 27% lower risk of stillbirth than unvaccinated women [48].

However, McMillan et al. (2015) reported that influenza vaccination during pregnancy was not associated with the risk of fetal death and spontaneous abortion, in agreement with our findings. No associations were found for congenital malformations in women vaccinated during their first trimester [49]. Polyzos et al. (2015) assessed the risk of congenital anomalies after influenza vaccination in 14 cohort studies and 1 case-control study. They also reported no association for major malformations or congenital defects, regardless of the trimester, agreeing with our study [50]. The most recent cohort study, conducted by Getahun et al. (2019) (n = 247,036) included the highest number of cases (n = 130,996), and reported that vaccination was associated with a decreased risk of stillbirth (OR: 0.88, 95% CI: 0.78–0.99), but no association was found for PTB and SGA[74]. Among the individual observational studies, PTB was the most frequently examined outcome; Chamber et al. (2013) reported an increased risk, whereas Olsen et al. (2016), Cleary et al. (2014) and Kallen et al. (2012) showed decreased risks. None of the studies showed increased risks of LBW, but Dodds et al. (2012), Kallen et al. (2012), and Legge et al. (2014) presented decreased risks of LBW. Fell et al. (2012) reported a decreased risk of SGA, and Regan et al. (2016), Fell et al. (2012) and Pasternak et al. (2012) showed significant (>50%) decreases in fetal death. However, none of the studies showed significant findings for congenital malformation.

In the LBW and SGA meta-regression analyses in our study, the significant summary estimates from cohort studies alone became null effects after adjusting for season at vaccination and income level of the country. These results might be attributable to temperature exposure (cold season) [75, 76] since seasonal influenza vaccination is usually administered during late fall to winter, along with nutritional and medical support for pregnancy outcomes [77].

A consistent null association between maternal influenza vaccination and adverse birth outcomes was confirmed in this study, and the need for a cautious interpretation of the beneficial birth outcomes reported by previous observational cohort studies was underscored [78]. The recent WHO report also concluded that the evidence for maternal immunization reducing adverse birth outcomes is conflicting. Numerous observational studies, as well as meta-analyses and systematic reviews including those observational studies, cannot escape the possibility of substantial bias, which is the main reason why the WHO report did not present a definitive statement on maternal immunization [55].

### Strengths and limitations

A major strength of this study is that it presents an up-to-date literature review, including the highest number of reasonable-quality articles. In addition, this study did not restrict the study design in the search step (only excluding cross-sectional studies), so we included RCTs, cohort studies, and case-control studies. We applied a Bayesian 3-level random-effects model that considered study design hierarchy to synthesize the adverse birth outcomes from different types of study design. Through this method, we obtained different results for adverse birth outcomes from the above-mentioned traditional meta-analyses [39, 47, 48], and compared the summary estimates across different study designs. Additionally, when we conducted a traditional meta-analysis comprising all cohort studies included in this analysis, the results were different from the Bayesian meta-analysis for cohort studies only. The traditional meta-analysis for cohort studies showed that the risks for PTB (OR = 0.91, 95% CI: 0.85–0.98) and fetal death (OR = 0.81, 95% CI: 0.72–0.90) were significantly reduced in the vaccinated group, but the risks for LBW(OR = 0.93, 95% CI: 0.87–1), SGA (OR = 0.97, 95% CI: 0.94–1.01) and congenital malformations (OR = 1.02, 95% CI: 0.97–1.09) were not (S6 Table). Our up-to-date Bayesian meta-analysis comprising only cohort studies presented significantly (P(OR<1) of over 90%) reduced risk for PTB, LBW, SGA, and fetal death, but after adjusting for season and income level as covariates, only fetal death remained significant. These results have the notable implication that different statistical models yield different results, so we should be cautious in interpreting the results.

Bayesian methods are becoming more common in a number of areas of healthcare research, including meta-analyses [58]. Using this type of model is highly advantageous for overcoming heterogeneity across studies. The advantages of Bayesian methods are that past experience or expert opinion can be used as prior information and the quantity of interest such as the credible interval (CrI) or the hypothesis can be directly interpreted as the probability based on the posterior distribution. In addition, Bayesian methods do not require a large sample size assumption, so they can provide accurate inferential results when the number of samples is small [79]. Furthermore, Bayesian methods can improve the precision by using a hierarchical model. However, in random-effects models, precision decreases with increasing heterogeneity, and confidence intervals widen correspondingly[80]. If other studies with different 3-level study designs, as used in our study, were to be included in a meta-analysis, the width of the resulting confidence interval might be increased in comparison to that of each individual study.

Although we tried to overcome study design heterogeneity by using Bayesian methods, this study did include many cohort studies, making it vulnerable to biases and making our study somewhat dependent on the outcomes from cohort studies. However, as explicitly stated throughout the Discussion section, when we compared our summary estimates to those of cohort studies, different conclusions were drawn. Therefore, our new Bayesian meta-analysis has novel implications. Another limitation is that incomplete information on vaccine type, the exact perinatal period, and the season when the vaccine was administered hindered us conducting a fully comprehensive covariate analysis; even though we gathered many variables in the primary data acquisition steps, we could not incorporate these variables fully in the meta-regression. The presence of few studies from low/middle-income countries is another limitation. Since few adverse birth outcomes occur in high-income countries, the small amount of data from low/middle-income countries cannot provide convincing evidence whether maternal influenza vaccination plans should be primarily implemented in low-resource countries. Post-marketing RCT studies might be conducted, as they have been suggested by the Food and Drug Administration as a maternal vaccination benefit-risk assessment tool when clinical trials are not appropriate before marketing authorization [81].

## Conclusion

In conclusion, this research presented additional evidence that maternal influenza vaccination is not associated with harmful effects on birth outcomes. The beneficial effects reported from previous studies on preterm birth, low birth weight, and fetal death could not be confirmed.

## Supporting information

S1 Table Characteristics of studies included in the Bayesian meta-analysis. (DOCX)

S2 Table Risk of bias assessment for included observational studies by the Newcastle-Ottawa Scale. (DOCX)

S3 Table Coefficients of meta-regression analyses on covariate effects. (DOCX)

S4 Table Information on the studies included for each birth outcome: (A) PTB, (B) LBW, (C) SGA, (D) congenital malformation, (E) fetal death. (DOCX)

S5 Table Meta-regression results on congenital malformation adjusted by gestational period when vaccination given. (DOCX)

S6 Table Summary estimates of conventional meta-analysis with random effects model constituted by cohort studies (A) PTB, (B) LBW, (C) SGA, (D) congenital malformation, (E) fetal death. (DOCX)

S1 Fig Risk of bias assessment for randomized controlled studies. (TIF)

S2 Fig Publication bias for included studies: (A) funnel plot of preterm birth, (B) funnel plot of low birth weight, (C) funnel plot of small for gestational age, (D) funnel plot of congenital malformation, (E) funnel plot of fetal death. (TIF)
