# Supplementary material for: Effects of maternal influenza vaccination on adverse birth outcomes: A systematic review and Bayesian meta-analysis
Source: PLoS One. 2019 Aug 14;14(8):e0220910. doi: 10.1371/journal.pone.0220910 (PMC6693758; doi:10.1371/journal.pone.0220910)
Supplement: S1 Table — (DOCX) [file pone.0220910.s001.docx]

S1 Table. Characteristics of studies included in Bayesian meta-analysis

| **Study** | **Design** | **Country** | **Income level** | **Period** | **Vaccine*** | **N (vaccinated**  **/unvaccinated )** | **Birth outcomes included in meta-analysis** |
| --- | --- | --- | --- | --- | --- | --- | --- |
| Steinhoff, 2017^[[1]](#endnote-1)^ | RCT | Nepal | LMI | seasonal (2011-2013) | TIV | 1,847/1,846 | PTB,LBW,SGA ,fetal death, congenital malformation |
| Madhi, 2014^[[2]](#endnote-2)^ | RCT | South Africa | LMI | seasonal (2011-2012) | TIV | 1,062/1,054 | PTB,LBW, fetal death |
| Chambers, 2013^[[3]](#endnote-3)^ | prospective cohort | USA | HI | pandemic (2009-2012) | H1N1 2009 or nonadjuvanted TIV | 831/191 | PTB, fetal death, congenital malformation |
| Chambers, 2016^[[4]](#endnote-4)^ | prospective cohort | USA | HI | seasonal (2010-2014) | NA | 1,263/467 | PTB,SGA, congenital malformation |
| Heikkinen, 2012^[[5]](#endnote-5)^ | prospective cohort | Netherlands | HI | pandemic (2009–2010) | MF59-adjuvanted A/H1N1 | 2,295/2,213 | PTB,LBW, fetal death, congenital malformation |
| Launay, 2012^[[6]](#endnote-6)^ | prospective cohort | France | HI | pandemic (2009-2010) | nonadjuvanted A/H1N1 2009 | 455/422 | congenital malformation |
| Ludvigsson, 2015^[[7]](#endnote-7)^ | prospective cohort | Sweden | HI | pandemic (2009-2010) | A(H1N1)pdm09 | 41,183/234,317 | fetal death |
| Ludvigsson, 2016^[[8]](#endnote-8)^ | prospective cohort | Sweden | HI | pandemic (2009-2011) | A(H1N1)pdm09 | 40,983/197,588 (infants and sibling) | congenital malformation |
| Ma, 2014^[[9]](#endnote-9)^ | prospective cohort | China | LMI | seasonal | split-virion nonadjuvanted A(H1N1) | 122/104 | PTB,LBW |
| Mackenzie, 2011^[[10]](#endnote-10)^ | prospective cohort | UK | HI | pandemic (2009-2010) | H1N1 2009 | 3,754/312 | congenital malformation |
| McHugh, 2019^[[11]](#endnote-11)^ | prospective cohort | Australia | HI | seasonal  (2012-2015) | inactivated influenza vaccine | 2,706/6,121 | PTB,LBW,SGA |
| Oppermann, 2012^[[12]](#endnote-12)^ | prospective cohort | Germany | HI | pandemic (2009-2010) | H1N1 2009 | 323/1,329 | PTB, congenital malformation |
| Adedinsewo,2013^[[13]](#endnote-13)^ | retrospective cohort | USA | HI | Seasonal  (2005-2008) | H1N1 | 916/4,506 | PTB,SGA |
| Ahrens, 2014^[[14]](#endnote-14)^ | retrospective cohort | USA | HI | seasonal (2014) | TIV | 637/1,499 | PTB,SGA |
| Arriola, 2017^[[15]](#endnote-15)^ | retrospective cohort | Nicaragua | LMI | seasonal | TIV | 1,789/1,479 | PTB,LBW,SGA |
| Baum, 2015^[[16]](#endnote-16)^ | retrospective cohort | Finland | HI | pandemic (2009) | AS03 adjuvanted split virion influenza A(H1N1)pdm09 | 34,214/9,363 | PTB,LBW,SGA |
| Beau,2014^[[17]](#endnote-17)^ | retrospective cohort | France | HI | seasonal (2009-2010) | nonadjuvant A/H1N1pdm09 | 1,645/3,290 | PTB, SGA, fetal death |
| Cantu, 2013^[[18]](#endnote-18)^ | retrospective cohort | USA | HI | both (2009-2010) | A/H1N1pdm09 | 1,094/2,010 | PTB,LBW,SGA ,fetal death |
| Cleary, 2014^[[19]](#endnote-19)^ | retrospective cohort | Ireland | HI | pandemic (2009-2010) | H1N1 2009  nonadjuvanted or AS03- adjuvanted | 2,996/3,898 | PTB, SGA, congenital malformation |
| Deinard, 1981^[[20]](#endnote-20)^ | prospective cohort | USA | HI | pandemic (1976-1977) | H1N1  nonadjuvanted | 186/489 | congenital malformation |
| Dodds, 2012^[[21]](#endnote-21)^ | retrospective cohort | Canada | HI | seasonal (2006-2009) | H1N1 | 1,925/7,722 | PTB,LBW,SGA |
| Fabiani, 2015^[[22]](#endnote-22)^ | retrospective cohort | Italy | HI | pandemic (2009-2010) | MF59-adjuvanted A/H1N1pdm09 | 2,048/100,029 | PTB,LBW, congenital malformation |
| Fell, 2012^[[23]](#endnote-23)^ | retrospective cohort | Canada | HI | pandemic (2009-2010) | H1N1pdm09 | 36,033/81,302  (infants) | PTB, SGA ,fetal death |
| Getahun, 2019^[[24]](#endnote-24)^ | retrospective cohort | USA | HI | seasonal  (2008-2016) | inactivated influenza vaccine | 130,996/116,040 | PTB, fetal death |
| Haberg,2013^[[25]](#endnote-25)^ | retrospective cohort | Norway | HI | pandemic (2009-2010) | AS03-adjuvanted or non-adjuvanted A/H1N1pdm09 | 25,105/21,386 | fetal death |
| Kallen, 2012^[[26]](#endnote-26)^ | retrospective cohort | Sweden | HI | Pandemic  (2009-2010) | H1N1 2009 pandemic, AS03-adjuvanted | 18,612/136,914 | PTB,LBW,SGA ,fetal death, congenital malformation |
| Kharbanda, 2017^[[27]](#endnote-27)^ | retrospective cohort | USA | HI | Seasonal  (2004-2013) | NA | 52,856/373,088  (infants) | congenital malformation |
| Legge, 2014^[[28]](#endnote-28)^ | retrospective cohort | Canada | HI | seasonal (2010-2012) | A/H1N1pdm09 | 1,958/10,265 | PTB,LBW,SGA |
| Lin, 2012^[[29]](#endnote-29)^ | retrospective cohort | Taiwan | LMI | Pandemic  (2009-2010) | H1N1 2009  nonadjuvanted | 198/198 | PTB,LBW, congenital malformation |
| Ludvigsson, 2013^[[30]](#endnote-30)^ | retrospective cohort | Sweden | HI | pandemic (2009-2010) | AS03-adjuvanted A/H1N1pdm09 | 13,297/7,790 | PTB,LBW,SGA |
| McHugh, 2017^[[31]](#endnote-31)^ | retrospective cohort | Australia | HI | seasonal (2012-2014) | NA | 2,429/4,697  (infants) | PTB,LBW |
| Munoz, 2005^[[32]](#endnote-32)^ | retrospective cohort | USA | HI | seasonal (1998-2003) | TIV nonadjuvanted | 252/826 | congenital malformation |
| Nordin, 2014^[[33]](#endnote-33)^ | retrospective cohort | USA | HI | seasonal (2005-2008) | TIV | 57,554/57,554 | PTB,SGA |
| Olsen, 2016^[[34]](#endnote-34)^ | prospective cohort | Laos | LMI | seasonal | TIV | 2,172/2,931 | PTB,SGA |
| Omer, 2011^[[35]](#endnote-35)^ | retrospective cohort | USA | HI | seasonal (2004-2006) | NA | 578/3,748 | PTB,SGA |
| Pasternak, 2012(1)^[[36]](#endnote-36)^ | retrospective cohort | Denmark | HI | pandemic | AS03-adjuvanted influenza A(H1N1)pdm09 | 7,014/43,633 | fetal death |
| Pasternak, 2012(2)^[[37]](#endnote-37)^ | retrospective cohort | Denmark | HI | pandemic (2009-2010) | AS03-adjuvanted influenza A/H1N1pdm09 | 6,989/46,443 | PTB,LBW, SGA, congenital malformation |
| Regan, 2016^[[38]](#endnote-38)^ | retrospective cohort | Australia | HI | seasonal | TIV | 3,169/27,859  (infants) | fetal death |
| Richards, 2013^[[39]](#endnote-39)^ | retrospective cohort | USA | HI | pandemic (2009-2010) | 2009 H1N1 or 2009–2010 TIV | 1,125/1,581 | SGA |
| Sheffield, 2012^[[40]](#endnote-40)^ | retrospective cohort | USA | HI | seasonal (2003-2008) | TIV .nonadjuvanted | 8,864/76,919 | congenital malformation |
| Trotta, 2014^[[41]](#endnote-41)^ | retrospective cohort | Italy | HI | pandemic (2009-2010) | A/H1N1 2009 MF59-adjuvanted | 6,131/23,987 | SGA, congenital malformation |
| Vazquez-Benitez, 2016^[[42]](#endnote-42)^ | retrospective cohort | USA | HI | both (2009-2010) | MIV | 18,469/28,080 | PTB,SGA |
| Zerbo, 2017^[[43]](#endnote-43)^ | retrospective cohort | USA | HI | seasonal | NA | 64,748/81,119 | PTB,LBW,SGA |
| Donahue, 2017^[[44]](#endnote-44)^ | Case-control study | USA | HI | Seasonal  (2010-2012) | A/California/7/2009 (H1N1)pdm09,  A/Perth/16/2009 (H3N2), B/Brisbane/60/2008 | 485/485 | Fetal death  (spontaneous abortion) |
| Irving, 2013^[[45]](#endnote-45)^ | Case-control study | USA | HI | seasonal (2005-2006) | TIV | 133/353 | fetal death |
| Louik, 2013^[[46]](#endnote-46)^ | Case-control study | USA | HI | pandemic (2009-2011) | unadjuvanted MIV or TIV | 1,524/2,341 | PTB |
| Louik, 2016^[[47]](#endnote-47)^ | Case-control study | USA | HI | seasonal (2011-2014) | H1N1 2009 or TIV | 774/1,136(infants) | PTB, congenital malformation |
| Sukumaran, 2018^[[48]](#endnote-48)^ | Case-control study | USA | HI | Both  (2004-2014) | NA | 25,222/25,222 | Mortality in the First 6 Months of Life |

*TIV: Trivalent Influenza Vaccine, MIV: Monovalent Influenza Vaccine

1. Steinhoff MC, Katz J, Englund JA, Khatry SK, Shrestha L, Kuypers J, et al. Year-round influenza immunisation during pregnancy in Nepal:a phase 4, randomised, placebo-controlled trial. Lancet Infect Dis. 2017;17(9):981-9. [↑](#endnote-ref-1)
2. Madhi SA, Cutland CL, Kuwanda L, Weinberg A, Hugo A, Jones S, et al. Influenza Vaccination of Pregnant Women and Protection of Their Infants. The New England Journal of Medicine. 2014;371:918-31. [↑](#endnote-ref-2)
3. Chambers CD, Johnson D, Xu R, Luo Y, Louik C, Mitchell AA, et al. Risks and safety of pandemic h1n1 influenza vaccine in pregnancy: Birth defects, spontaneous abortion, preterm delivery, and small for gestational age infants. Vaccine.2013:31; 5026-5032 [↑](#endnote-ref-3)
4. Chambers CD, Johnson DL, Xub R, Luo YJ, Louik C, Mitchell AA, et al. Safety of the 2010–11, 2011–12, 2012–13, and 2013–14 seasonal influenza vaccines in pregnancy: Birth defects, spontaneous abortion, preterm delivery, and small for gestational age infants, a study from the cohort arm of VAMPSS. Vaccine. 2016;34:4443-9. [↑](#endnote-ref-4)
5. Heikkinen T, Young J, Beek Ev, Franke H, Verstraeten T, Weil JG, et al. Safety of MF59-adjuvanted A/H1N1 influenza vaccine in pregnancy: a comparative cohort study. American Journal of Obstetrics & Gynecology. 2012; 207:177.e1-.e8. [↑](#endnote-ref-5)
6. Launay O, Krivine A, Charlier C, Truster V, Tsatsaris V, Lepercq J, et al. Low Rate of Pandemic A/H1N1 2009 Influenza Infection and Lack of Severe Complication of Vaccination in Pregnant Women: A Prospective Cohort Study. PLOS ONE. 2012 ; 7(12): e52303. [↑](#endnote-ref-6)
7. Ludvigsson JF, Ström P, Lundholm C, Cnattingius S, Ekbom A, Örtqvist Å, et al. Maternal vaccination against H1N1 influenza and offspring mortality: population based cohort study and sibling design. BMJ. 2015; 351(h5585):1-6. [↑](#endnote-ref-7)
8. Ludvigsson JF, Strom P, Lundholm C, Cnattingius S, Ekbom A, Ortqvist Å, et al. Risk for Congenital Malformation With H1N1Influenza Vaccine: A Cohort Study With Sibling Analysis. American College of Physicians. 2016;165:848-55. [↑](#endnote-ref-8)
9. Ma F, Zhang L, Jiang R, Zhang J, Wang H, Gao X, et al. Prospective Cohort Study of the Safety of an Influenza A(H1N1) Vaccine in Pregnant Chinese Women. Clinical and Vaccine Immunology.2014;21(9):1282-1287. [↑](#endnote-ref-9)
10. Mackenzie IS, MacDonald TM, Shakir S, Dryburgh M, Mantay BJ, McDonnell P, et al. Influenza H1N1 (swine flu) vaccination: a safety surveillance feasibility study using self-reporting of serious adverse events and pregnancy outcomes. British Journal of Clinical Pharmacology. 2011; 73 (5):801-11. [↑](#endnote-ref-10)
11. Lisa McHugh, Helen S. Marshall, Kirsten P. Perrett, Terry Nolan, Nicholas Wood, Stephen B. Lambert, et al. The Safety of Influenza and Pertussis Vaccination in Pregnancy in a Cohort of Australian Mother-Infant Pairs,2012–2015: The FluMum Study. CID 2019:68 (1 February) [↑](#endnote-ref-11)
12. Oppermanna M, Fritzsche J, Weber-Schoendorfer C, Keller-Stanislawski B, Allignol A, Meister R, Schaefer C, et al. A(H1N1)v2009: A controlled observational prospective cohort study on vaccine safety in pregnancy.Vaccine.2012;30:4445-4452. [↑](#endnote-ref-12)
13. Adedinsewo DA, Noory L, Bednarczyk RA, Steinhoff MC, Davis R, Ogbuanu C, et al. Impact of maternal characteristics on the effect of maternal influenzavaccination on fetal outcomes. Vaccine.2013;31:5827-5833. [↑](#endnote-ref-13)
14. Ahrens KA, Louik C, Kerr S, Mitchell AA, Werler MM. Seasonal Influenza Vaccination during Pregnancy and the Risks of Preterm Delivery and Small for Gestational Age Birth. Paediatr Perinat Epidemiol. 2014;28(6):498-509. [↑](#endnote-ref-14)
15. Arriola CS, Vascone N, Thompson MG, Olsen SJ, Moen AC, Bresee J, et al. Association of influenza vaccination during pregnancy with birth outcomes in Nicaragua.Vaccine.2017;35:3056-3063. [↑](#endnote-ref-15)
16. Baum U, Leino T, Gissler M, Kilpi T, Jokinen J. Perinatal survival and health after maternal influenza A (H1N1)pdm09 vaccination: A cohort study of pregnancies stratified by trimester of vaccination. Vaccine. 2015; 33:4850-7. [↑](#endnote-ref-16)
17. Beau AB, Hurault-Delarue C, Vidal S, Guitard C, Vayssière C, Petiot D, et al. Pandemic A/H1N1 influenza vaccination during pregnancy: A comparative study using the EFEMERIS database. Vaccine. 2014;32:1254-8. [↑](#endnote-ref-17)
18. Cantu J, Biggio J, Jauk V, Wetta L, Andrews W, Tita A. Selective uptake of influenza vaccine and pregnancy outcomes. The Journal of Maternal-Fetal & Neonatal Medicine. 2013. 26:12; 1207-1211. [↑](#endnote-ref-18)
19. Cleary BJ, Rice Ú, Eogan M, Metwally N, McAuliffe F. 2009 A/H1N1 influenza vaccination in pregnancy: uptake and pregnancy outcomes – a historical cohort study. European Journal of Obstetrics & Gynecology and Reproductive Biology. 2014; 178:163-8. [↑](#endnote-ref-19)
20. Deinard AS, Ogburn P Jr. A/NJ/8/76 influenza vaccination program: effects on maternal health and pregnancy outcome. [Am J Obstet Gynecol.](https://www.ncbi.nlm.nih.gov/pubmed/7246624) 1981 Jun 1;140(3):240-5. [↑](#endnote-ref-20)
21. Dodds L, MacDonald N, Scott J, Spencer A, Allen VM, McNeil S. The Association Between Influenza Vaccine in Pregnancy and Adverse Neonatal Outcomes. J Obstet Gynaecol Can. 2012; 34(8):714-20. [↑](#endnote-ref-21)
22. Fabiani M, Bella A, Rota MC, Clagnan E, Gallo T, D’Amato M, et al. A/H1N1 pandemic influenza vaccination: A retrospective evaluation of adverse maternal, fetal and neonatal outcomes in a cohort of pregnant women in Italy.2015.Vaccine:33; 2240-2247. [↑](#endnote-ref-22)
23. Fell DB, Sprague AE, Liu N, Yasseen III AS, Wen SW, Smith G, et al. H1N1 Influenza Vaccination During Pregnancy and Fetal and Neonatal Outcomes. American Journal of Public Health.2012:102(6); e33-e40. [↑](#endnote-ref-23)
24. Getahun D, Fassett MJ, Peltier MR,Takhar HS,Shaw SF, Im TM, et al. Association between seasonal influenza vaccination with pre- and postnatal outcomes.Vaccine. 2019: https://doi.org/10.1016/j.vaccine.2019.02.019. [↑](#endnote-ref-24)
25. Håberg SE, Trogstad L, Gunnes N, Wilcox AJ, Gjessing HK, Samuelsen SO, et al. Risk of Fetal Death after Pandemic Influenza Virus Infection or Vaccination. N Engl J Med.2013: 368;4. [↑](#endnote-ref-25)
26. Kallen B, Olausson P. Vaccination against H1N1 influenza with Pandemrix during pregnancy and delivery outcome: a Swedish register study. BJOG. 2012;119: 1583-90. [↑](#endnote-ref-26)
27. Kharbanda EO, Vazquez-Benitez G, Romitti PA, Naleway AL, Cheetham TC, Lipkind HS, et al. First Trimester Influenza Vaccination and Risks for Major Structural Birth Defects in Offspring. The Journal of Pediatrics. 2017; 187: 234-9.e4. [↑](#endnote-ref-27)
28. Legge A, Dodds L, MacDonald NE, Scott J, McNeil S. Rates and determinants of seasonal influenza vaccination in pregnancy and association with neonatal outcomes. CMAJ. 2014:186(4):E157-E64. [↑](#endnote-ref-28)
29. Lin TH, Lin SY, Lin CH, Lin RI, Lin HC, Chiu TH, et al. AdimFlu-S® influenza A (H1N1) vaccine during pregnancy: The Taiwanese

    Pharmacovigilance Survey. Vaccine. 2012: 30; 2671-2675. [↑](#endnote-ref-29)
30. Ludvigsson JF, Zugna D, Cnattingius S, Richiardi L, Ekbom A, Ortqvist A, et al. Influenza H1N1 vaccination and adverse pregnancy outcome. Eur J Epidemiol. 2013:28;579-88. [↑](#endnote-ref-30)
31. McHugh L, Andrews RM, Lambert SB, Viney KA, Wood N, Perrett KP, et al. Birth outcomes for Australian mother-infant pairs who received an influenza vaccine during pregnancy, 2012–2014: The FluMum study. Vaccine. 2017:35;1403-1409. [↑](#endnote-ref-31)
32. Munoz FM, Greisinger AJ, Wehmanen OA, Mouzoon ME, Hoyle JC, Smith FA, et al. Safety of influenza vaccination during pregnancy. American Journal of Obstetrics and Gynecology.2005: 192;1098–106. [↑](#endnote-ref-32)
33. Nordin JD, Kharbanda EO, Benitez GV, Lipkind H, Vellozzi C, DeStefano F, et al. Maternal Influenza Vaccine and Risks for Preterm or Small for Gestational Age Birth. THE JOURNAL OF PEDIATRICS. 2014;164(5):1051-7. [↑](#endnote-ref-33)
34. Olsen SJ, Mirza SA, Vonglokham P, Khanthamaly V, Chitry B, Pholsena V, et al. The Effect of Influenza Vaccination on Birth Outcomes in a Cohort of Pregnant Women in Lao PDR, 2014–2015. Clin Infect Dis. 2016: 63(4); 487–494. [↑](#endnote-ref-34)
35. Omer SB, Goodman D, Steinhoff MC, Rochat R, Klugman KP, Stoll BJ, et al. Maternal Influenza Immunization and Reduced Likelihood of Prematurity and Small for Gestational Age Births: A Retrospective Cohort Study. PLoS Medicine. 2011; 8(5):e1000441. [↑](#endnote-ref-35)
36. Pasternak B, Svanstrom H, Mølgaard-Nielsen D, Krause TG, Emborg H-D, Melbye M, et al. Vaccination against pandemic A/H1N1 2009 influenza in pregnancy and risk of fetal death: cohort study in Denmark. BMJ. 2012: 344; e2794 doi: 10.1136/bmj.e2794. [↑](#endnote-ref-36)
37. Pasternak B, Svanstrom H, Mølgaard-Nielsen D, Krause TG, Emborg HD, Melbye M, et al. Risk of Adverse Fetal Outcomes Following Administration of a Pandemic Influenza A(H1N1) Vaccine During Pregnancy. JAMA.2012: 308(2); 165-174. [↑](#endnote-ref-37)
38. Regan AK, Moore HC, de Klerk N, Omer SB, Shellam G, Mak DB, et al. Seasonal Trivalent Influenza Vaccination During Pregnancy and the Incidence of Stillbirth: Population-Based Retrospective Cohort Study. Clinical Infectious Diseases. 2016: 62(10); 1221-1227 [↑](#endnote-ref-38)
39. Richards JL, Hansen C, Bredfeldt C, Bednarczyk RA, Steinhoff MC, Adjaye-Gbewonyo D, et al. Neonatal Outcomes After Antenatal Influenza Immunization During the 2009 H1N1 Influenza Pandemic: Impact on Preterm Birth, Birth Weight, and Small for Gestational Age Birth. Clinical Infectious Diseases. 2013:56(9); 1216-1222. [↑](#endnote-ref-39)
40. Sheffield JS, Greer LG, Rogers VL, Roberts SW, Lytle H, McIntire DD, et al. Effect of Influenza Vaccination in the First Trimester of Pregnancy. Obstet Gynecol. 2012;120:532-7. doi: http://10.1097/AOG.0b013e318263a278. [↑](#endnote-ref-40)
41. Trotta F, Da Cas R, Gramegna M, Zocchetti C, Alegiani SS, Venegoni M, et al. Evaluation of safety of A/H1N1 pandemic vaccination during pregnancy: cohort study. BMJ. 2014: 348; g3361. [↑](#endnote-ref-41)
42. Vazquez-Benitez G, Kharbanda EO, Naleway AL, Lipkind H, Sukumaran L, McCarthy NL, et al. Risk of Preterm or Small-for-Gestational-Age Birth After Influenza Vaccination During Pregnancy: Caveats When Conducting Retrospective Observational Studies. Am J Epidemiol. 2016: 184(3);176-86. doi: 10.1093/aje/kww043. [↑](#endnote-ref-42)
43. Zerbo O, Modaressi S, Chan B, Goddard K, Lewis N, Bok K, et al. No association between influenza vaccination during pregnancy and adverse birth outcomes. No association between influenza vaccination during pregnancy and adverse birth outcomes Vaccine. 2017:35;3186-3190. [↑](#endnote-ref-43)
44. James G. Donahue JG, Kieke BA, King JP, DeStefano F, Mascola MA, Irving SA, et al. Association of spontaneous abortion with receipt of inactivated influenza vaccine containing H1N1pdm09 in 2010–11 and 2011–12. Vaccine. 2017: 35; 5314-5322 [↑](#endnote-ref-44)
45. Irving SA, Kieke BA, Donahue JG, Mascola MA, Baggs J, DeStefano F, et al. Trivalent Inactivated Influenza Vaccine and Spontaneous Abortion. OBSTETRICS & GYNECOLOGY. 2013:121(1); 159-165 [↑](#endnote-ref-45)
46. Louik C, Ahrens K, Kerr S, Pyo J, Chambers C, Jones KL, et al. Risks and safety of pandemic H1N1 influenza vaccine in pregnancy: Exposure prevalence, preterm delivery, and specific birth defects.Vaccine.2013: 31; 5033-5040. [↑](#endnote-ref-46)
47. Louik C, Kerr S, Bennekom CMV, Chambers C, Jones KL, Schatz M, et al. Safety of the 2011–12, 2012–13, and 2013–14 seasonal influenza vaccines in pregnancy: Preterm delivery and specific malformations, a study from the case-control arm of VAMPSS. Vaccine. 2016;34: 4450-4459. [↑](#endnote-ref-47)
48. Sukumaran L, McCarthy NL, Kharbanda EO, Vazquez-Benitez G, Lipkind HS, Jackson L, et al. Infant Hospitalizations and Mortality After Maternal Vaccination. PEDIATRICS. 2018:141(3); e20173310. [↑](#endnote-ref-48)
