# Supplementary material for: Effects of maternal influenza vaccination on adverse birth outcomes: A systematic review and Bayesian meta-analysis
Source: PLoS One. 2019 Aug 14;14(8):e0220910. doi: 10.1371/journal.pone.0220910 (PMC6693758; doi:10.1371/journal.pone.0220910)
Supplement: S2 Table — (DOCX) [file pone.0220910.s002.docx]

| **Quality assessment criteria** | **Acceptable** | Chambers  2013 | Chambers  2016 | Heikkinen  2012 | Launay  2012 | Ludvig-  sson  2015 | Ludvig-  sson  2016 | Ma, 2014 | McHugh  2019 | Macken-  zie  2011 | Opper-  mann  2012 | Adedin-  sewo  2013 |
| --- | --- | --- | --- | --- | --- | --- | --- | --- | --- | --- | --- | --- |
| ***Selection*** |  |  |  |  |  |  |  |  |  |  |  |  |
| Representativeness of exposed cohort? | Representative of average adult in community (age/sex/being at risk of disease) | O | O | O | O | O | O | O | O | O | O | O |
| Selection of the non-exposed cohort? | Drawn from same community as exposed cohort | O | O | O | O | O | O | O | O | O | O | O |
| Is the Case Definition Adequate? | Yes, with independent validation | O | O | O | O | O | O | O | O | O | O | O |
| Definition of Controls? | NO history of vaccination | O | O | O | O | O | O | O | O | O | O | O |
| ***Comparability*** |  |  |  |  |  |  |  |  |  |  |  |  |
| Study controls for age/sex? | Yes | O | O | O | O | O | O | O | O | O | O | O |
| Study controls for at least 3 additional risk factors? | Gestational age, insurance type,  co- morbidities (asthma, chronic  hypertension diabetes mellitus) | O | O | O | O | O | O | O | O | O | O | O |
| ***Outcome*** |  |  |  |  |  |  |  |  |  |  |  |  |
| Adequacy of follow-up of cohorts? | Complete follow-up, or subjects lost to follow-up unlikely to introduce bias | O | O | O | O | O | O | O | O | O | O | O |
| Assessment of outcome? | Independent blind assessment, record linkage | NA | NA | NA | NA | NA | NA | NA | X | NA | NA | NA |
|  | **Final standards** | Good | Good | Good | Good | Good | Good | Good | Good | Good | Good | Good |

# S2 Table. Risk of bias assessment for included observational studies by Newcastle-Ottawa Scale

| **Quality assessment criteria** | **Acceptable** | Ahrens 2014 | Arriola 2017 | Baum  2015 | Beau  2014 | Cantu 2013 | Cleary 2014 | Deinard 1981 | Dodds 2012 | Fabiani 2015 | Getahun  2019 | Haberg  2013 | Kallen 2012 |
| --- | --- | --- | --- | --- | --- | --- | --- | --- | --- | --- | --- | --- | --- |
| ***Selection*** |  |  |  |  |  |  |  |  |  |  |  |  |  |
| Representativeness of exposed cohort? | Representative of average adult in community (age/sex/being at risk of disease) | O | O | O | O | O | O | O | O | O | O | O | O |
| Selection of the non-exposed cohort? | Drawn from same community as exposed cohort | O | O | O | O | O | O | O | O | O | O | O | O |
| Is the Case Definition Adequate? | Yes, with independent validation | O | O | O | O | O | O | O | O | O | O | O | O |
| Definition of Controls? | NO history of vaccination | O | O | O | O | O | O | O | O | O | O | X | O |
| ***Comparability*** |  |  |  |  |  |  |  |  |  |  |  |  |  |
| Study controls for age/sex? | Yes | O | O | O | O | O | O | O | O | O | O | O | O |
| Study controls for at least 3 additional risk factors? | Gestational age, insurance type,  co- morbidities (asthma, chronic  hypertension diabetes mellitus) | O | O | O | O | O | O | O | O | O | O | X | O |
| ***Outcome*** |  |  |  |  |  |  |  |  |  |  |  |  |  |
| Adequacy of follow-up of cohorts? | Complete follow-up, or subjects lost to follow-up unlikely to introduce bias | O | O | O | O | O | O | O | O | O | O | O | O |
| Assessment of outcome? | Independent blind assessment, record linkage | NA | NA | NA | NA | NA | NA | NA | NA | NA | O | O | O |
|  | **Final standards** | Good | Good | Good | Good | Good | Good | Good | Good | Good | Good | Fair | Good |

| **Quality assessment criteria** | **Acceptable** | Kharban-da 2017 | Legge 2014 | Lin  2012 | Ludvig-  sson 2013 | Mc-Hugh  2017 | Munoz  2005 | Nordin  2014 | Olsen  2016 | Omer  2011 | Paster-  Nak  2012(1) | Paster-  Nak  2012(2) | Regan 2016 |
| --- | --- | --- | --- | --- | --- | --- | --- | --- | --- | --- | --- | --- | --- |
| ***Selection*** |  |  |  |  |  |  |  |  |  |  |  |  |  |
| Representativeness of exposed cohort? | Representative of average adult in community (age/sex/being at risk of disease) | O | O | O | O | O | O | O | O | O | O | O | O |
| Selection of the non-exposed cohort? | Drawn from same community as exposed cohort | O | O | O | O | O | O | O | O | O | O | O | O |
| Is the Case Definition Adequate? | Yes, with independent validation | O | O | O | O | O | O | O | O | O | O | O | O |
| Definition of Controls? | NO history of vaccination | O | O | O | O | O | O | O | O | O | O | O | O |
| ***Comparability*** |  |  |  |  |  |  |  |  |  |  |  |  |  |
| Study controls for age/sex? | Yes | O | O | X | O | X | X | X | O | O | X | O | O |
| Study controls for at least 3 additional risk factors? | Gestational age, insurance type,  co- morbidities  (asthma, chronic  hypertension diabetes mellitus) | O | O | X | O | X | X | X | X | X | X | O | O |
| ***Outcome*** |  |  |  |  |  |  |  |  |  |  |  |  |  |
| Adequacy of follow-up of cohorts? | Complete follow-up, or subjects lost to follow-up unlikely to introduce bias | O | O | O | O | O | O | O | O | O | O | O | O |
| Assessment of outcome? | Independent blind assessment, record linkage | O | O | X | O | X | X | X | X | O | O | O | O |
|  | **Final standards** | Good | Good | Fair | Good | Fair | Fair | Fair | Fair | Fair | Fair | Good | Good |

| **Quality assessment criteria** | **Acceptable** | Richar-  ds 2013 | Sheffiel-d 2012 | Trotta  2014 | Vazquez-Benitez  2016 | Zerbo  2017 | Dona-  hue  2017 | Irving  2013 | Louik  2013 | Louik  2016 | Sukumuran  2018 |
| --- | --- | --- | --- | --- | --- | --- | --- | --- | --- | --- | --- |
| ***Selection*** |  |  |  |  |  |  |  |  |  |  |  |
| Representativeness of exposed cohort? | Representative of average adult in community (age/sex/being at risk of disease) | O | O | O | O | O | O | O | O | O | O |
| Selection of the non-exposed cohort? | Drawn from same community as exposed cohort | O | O | O | O | O | O | O | O | O | O |
| Is the Case Definition Adequate? | Yes, with independent validation | O | O | O | O | O | O | O | O | O | O |
| Definition of Controls? | NO history of vaccination | O | O | O | O | O | O | O | O | O | O |
| ***Comparability*** |  |  |  |  |  |  |  |  |  |  |  |
| Study controls for age/sex? | Yes | X | O | O | X | O | O | X | O | O | O |
| Study controls for at least 3 additional risk factors? | Gestational age, insurance type,  co- morbidities (asthma, chronic  hypertension diabetes mellitus) | O | X | O | O | O | O | O | O | O | O |
| ***Outcome*** |  |  |  |  |  |  |  |  |  |  |  |
| Adequacy of follow-up of cohorts? | Complete follow-up, or subjects lost to follow-up unlikely to introduce bias | O | O | O | O | O | O | O | O | X | O |
| Assessment of outcome? | Independent blind assessment, record linkage | O | O | O | O | O | O | O | O | X | O |
|  | **Final standards** | Good | Good | Good | Good | Good | Good | Good | Good | Fair | Good |

Pasternak, 2012 (1): published in BMJ, Pasternak, 2012(2): published in JAMA

***Thresholds for converting the Newcastle-Ottawa scales to AHRQ standards (good, fair, and poor):^[[1]](#footnote-1)^

Good quality: 3 or 4 stars in selection domain AND 1 or 2 stars in comparability domain AND 2 or 3 stars in outcome/exposure domain

Fair quality: 2 stars in selection domain AND 1 or 2 stars in comparability domain AND 2 or 3 stars in outcome/exposure domain

Poor quality: 0 or 1 star in selection domain OR 0 stars in comparability domain OR 0 or 1 stars in outcome/exposure domain

1. https://www.ncbi.nlm.nih.gov/books/NBK100793/bin/appd-fm1.pdf [↑](#footnote-ref-1)
