# Supplementary material for: Effects of maternal influenza vaccination on adverse birth outcomes: A systematic review and Bayesian meta-analysis
Source: PLoS One. 2019 Aug 14;14(8):e0220910. doi: 10.1371/journal.pone.0220910 (PMC6693758; doi:10.1371/journal.pone.0220910)
Supplement: S3 Table — (DOCX) [file pone.0220910.s003.docx]

S3 Table. Coefficients (beta) of covariate effects by meta-regression analyses

| Birth Outcome | Covariates | | Regression coefficient (β) | | | |
| --- | --- | --- | --- | --- | --- | --- |
|  |  |  | Median β | 95% Credible interval | | Probability (β >0) |
| Preterm birth | Vaccination time at season | seasonal vaccination | 0.114 | -0.123 | 0.376 | 0.823 |
|  |  | pandemic vaccination | 0.142 | -0.074 | 0.380 | 0.903 |
|  | Income level | **HI#** | -0.141 | -0.276 | -0.004 | 0.022 |
| Low birth weight | Vaccination time at season | seasonal vaccination | 0.645 | -1.326 | 2.631 | 0.715 |
|  |  | pandemic vaccination | 0.540 | -1.428 | 2.517 | 0.678 |
|  | Income level | HI | -0.042 | -0.243 | 0.180 | 0.335 |
| Small for gestational age | Vaccination time at season | seasonal vaccination | -0.172 | -0.615 | 0.242 | 0.209 |
|  |  | pandemic vaccination | -0.202 | -0.646 | 0.208 | 0.171 |
|  | Income level | HI | -0.011 | -0.102 | 0.075 | 0.394 |
| Congenital malformation | Vaccination time at pregnancy | 1st | 0.013 | -0.183 | 0.185 | 0.567 |
|  |  | 2nd-3rd | 0.144 | -0.095 | 0.372 | 0.902 |
|  | Vaccination time at season | seasonal vaccination | -0.290 | -0.661 | 0.058 | 0.050 |
|  |  | pandemic vaccination | -0.282 | -0.662 | 0.075 | 0.059 |
|  | Income level | HI | 0.2025 | -0.8854 | 1.5820 | 0.660 |
| Fetal death | Vaccination time at season | seasonal vaccination | 0.289 | -0.217 | 0.765 | 0.881 |
|  |  | pandemic vaccination | 0.027 | -0.406 | 0.433 | 0.556 |
|  | Income level | HI | -0.067 | -0.354 | 0.242 | 0.305 |

*Abbreviation: HI, high income; LMI, Lower Middle Income

**Reference category: Vaccination time at pregnancy (Total period), Vaccination time at season

(both seasonal and pandemic), Income (LMI)

#significant result
