# Supplementary material for: Effects of maternal influenza vaccination on adverse birth outcomes: A systematic review and Bayesian meta-analysis
Source: PLoS One. 2019 Aug 14;14(8):e0220910. doi: 10.1371/journal.pone.0220910 (PMC6693758; doi:10.1371/journal.pone.0220910)
Supplement: S4 Table — (DOCX) [file pone.0220910.s004.docx]

**S4(A) Table. Information of studies included in preterm birth (PTB) analysis**

| **Name** | **No. vaccinated (event)** | **No. Unvaccinated (event)** | **Effect size**  **95% CI** | **Definition of PTB** |
| --- | --- | --- | --- | --- |
| Steinhoff, 2017 | 1,809(225) * | 1,813(247)* | 0.91(RR)**  0.77-1.08 | <37ws |
| Madhi, 2014 | 1,062(108) | 1,054(96) | 1.12(OR)  0.837-1.489 | <37ws |
| Getahun, 2019 | 130,996(9234) | 116,040(9930) | 1.02(aHR)  0.99-1.05 | <37ws |
| McHugh,2019 | 2,706(184) | 6,086(406) | 1.10(HR)  0.92-1.31 | <37ws |
| Arriola, 2017 | 1,789(95) | 1,479(80) | 0.88(OR)  0.73-1.06 | <37ws |
| Zerbo, 2017 | 64,748(4183) | 81,119(6740) | 0.69(OR)  0.66-0.72 | <37ws |
| McHugh, 2017 | 2,397(436) | 4,718(754) | 1.14(RR)**  0.12-1.27 | <37ws |
| Chambers, 2016 | 1,092(72) | 379(21) | 1.23(HR)  0.75-2.02 | <37ws |
| Vazquez-Benitez, 2016 | 19,157(NA) | 27,392(NA) | 0.91(RR)¶  0.83-1.00 | >22ws, <37ws, |
| Olsen, 2016 | 1,819(136) | 1,890(242) | **0.70(RR)****  **0.57-0.87** | <37ws |
| Baum, 2015 | 34,241(1267) | 9,363(370) | 1.00(HR)  0.89-1.12 | ≥28,<37w |
| Fabiani,2015 | 135,174(110)*** | 10,286,489 (5531)*** | 1.15(HR)  0.95-1.39 | <37ws |
| Ma, 2014 | 122(0) | 101(1) | NA | NA |
| Ahrens, 2014 | 310(24) | 128(83) | 1.03(OR)  0.83-2.05 | <37ws |
| Beau,2014 | 1,522(93) | 2,890(216) | 0.82(HR)  0.64-1.06 | <37ws |
| Cleary, 2014 | 2,996(141) | 3,898(252) | **0.71(OR)**  **0.58-0.88** | <37ws |
| Legge, 2014 | 1,856(92) | 9,437(617) | 0.75(OR)  0.60-0.94 | <37ws |
| Nordin, 2014 | 57,554(3390) | 57,554(3478) | 0.97(OR)  0.93-1.02 | <37ws |
| Adedinsewo,2013 | NA | NA | 0.83(OR)  0.60-1.17 | <37ws |
| Cantu, 2013 | 979(126) | 2,008(194) | 1.2(RR)**  0.9-1.6 | <37ws |
| Ludvigsson,2013 | 13,297(635) | 7,790(456) | 0.99(OR)  0.89–1.10 | <37ws |
| Chambers, 2013 | 736(69) | 157(6) | **3.28(HR)**  **1.25-8.63** | <37ws |
| Heikkinen, 2012 | 2,295(84) | 2,213(108) | 0.75(OR)  0.55-0.99 | NA |
| Oppermann, 2012 | 323(29) | 1,329(122) | 0.98(OR)  0.64-1.49 | <37ws |
| Dodds,2012 | 1,925(124) | 7,722(584) | 0.84(RR)**  0.69-1.02 | <37ws |
| Fell, 2012 | 23,280(1376) | 32,091(2066) | 0.95(RR)**  0.88-1.02 | <37ws |
| Kallen,2012 | 18,612(592) | 136,914(4017) | **0.86(OR)**  **0.77-0.96** | <37ws |
| Lin,2012 | 202(14) | 206(25) | 0.54(OR)  0.27-1.07 | <37ws |
| Pasternak, 2012(2)_1# | 330(31) | 330(25) | 1.32(OR)##  0.76-2.31 | <37ws |
| Pasternak, 2012(2)_2$ | 6,642(302) | 6,642(295) | 1(OR)##  0.84-1.17 | <37ws |
| Omer, 2011 | 578(NA) | 3,590(NA) | 0.83(OR)  0.55–1.26 | <37ws |
| Louik, 2016 | 774(44) | 1,136(63) | 1.22(HR)  0.79-1.88 | <37ws |
| Louik, 2013 | 378(21) | 573(23) | 1.03(HR)  0.5-2.10 | <37ws |

*PY: Person Year

**RR: Risk Ratio

¶RR: Relative risk

***PD: pregnant days at risk

# vaccine administration during the 1st trimester

## POR: Prevalence Odds Ratio

$ vaccine administration during the 2nd-3rd trimester

**S4(B) Table. Information of studies included in low birth weight (LBW) analysis**

| **Name** | **No. vaccinated (event)** | **No. Unvaccinated (event)** | **Effect size,**  **95% CI** | **Definition of LBW** |
| --- | --- | --- | --- | --- |
| Steinhoff, 2017 | 1,380(315)* | 1,361(365)* | **0.85(RR)****  **0.75-0.97** | <2500g |
| Madhi, 2014 | 1,044(133) | 1,037(122) | N/A | <2500g |
| McHugh,2019 | 2,506(55) | 5,680(126) | 1.05(HR)  0.76-1.44 | <2500g |
| Arriola, 2017 | 1,789(99) | 1,497(96) | 0.83(OR)  0.62-1.06 | <2500g |
| McHugh, 2017 | 2,385(146) | 4,696(277) | 1.04(RR)**  0.85-1.26 | 1500 ≥, <2500g |
| Zerbo, 2017 | 64,748(3242) | 81,119(5128) | 0.95(HR)  0.86-1.06 | 1500 ≥, <2500g |
| Baum, 2015 | 34,241(828) | 9,363(236) | 1.05(HR)  0.90-1.21 | 1500 ≥, <2500g |
| Fabiani, 2015 | 28,523(47)*** | 1,437,487  (2624)*** | 0.92(HR)  0.69-1.23 | <2500g |
| Ma, 2014 | 122(2) | 104(1) | 1.70(OR)  0.152-19.07 | N/A |
| Legge, 2014 | 1,856(65) | 9,437(461) | **0.73(OR)**  **0.56-0.95** | <2500g |
| Cantu, 2013 | 979(98) | 2,010(178) | 1.0(RR)**  0.8-1.5 | <2500g |
| Ludvigsson, 2013 | 13,297(337) | 7,790(301) | 0.91(OR)  0.79-1.04 | <2500g |
| Heikkinen, 2012 | 2,295(64) | 2,213(68) | 0.88(OR)  0.61-1.26 | N/A |
| Lin, 2012 | 202(16) | 206(28) | 0.583(OR)  0.301-1.11 | <2500g |
| Dodds, 2012 | 1,925(76) | 7,722(427) | **0.74(RR)****  **0.58-0.95** | <2500g |
| Kallen, 2012 | 18,844(382) | 138,931(4416) | **0.86(OR)**  **0.77-0.96** | <2500g |
| Pasternak, 2012(2)_1^#^ | 330(15) | 330(18) | 0.83(OR)##  0.41-1.67 | <2500g |
| Pasternak,  2012(2)_2$ | 6,642(225) | 6,642(199) | 1.14(OR)##  0.94-1.38 | <2500g |

*PY: Person Year

**RR: Risk Ratio

***PD: Pregnant days at risk

# vaccine administration during the 1^st^ trimester

## POR: Prevalence Odds Ratio

$ vaccine administration during the 2^nd^-3rd trimester

**S4 (C) Table. Information of studies included in small for gestational age (SGA) analysis**

| **Name** | **No. vaccinated (event)** | **No. Unvaccinated (event)** | **Effect size**  **95% CI** | **Definition of SGA** |
| --- | --- | --- | --- | --- |
| Steinhoff, 2017 | 1,300(638)* | 1,307(538)* | 0.95(RR)**  0.87-1.04 | <10% |
| Getahun, 2019 | 130,996  (13,040) | 116,040  (11,212) | 0.99(aHR)  0.96-1.02 | <10% |
| McHugh,2019 | 2,687(255) | 6,082(609) | 0.99(HR)  0.86-1.15 | <10% |
| Arriola, 2017 | 2,223(NA) | 3,688(NA) | 1.02(OR)  0.83-1.24 | <10% |
| Zerbo, 2017 | 64,748(3,437) | 81,119(4,300) | 0.98(OR)  0.92-1.04 | <10% |
| Chambers, 2016 | 1,154(87) | 395(20) | 1.49(RR)¶  0.93-2.39 | <10% |
| Olsen, 2016 | 1,811(85) | 1,888(71) | 1.25(RR)**  0.91-1.72 | <10% |
| Vazquez-Benitez, 2016 | 19,157(830) | 27,392(860) | 1.0(RR)¶  0.91-1.03 | <10% |
| Baum, 2015 | 34,241(627) | 9,363(148) | 1.17(OR)  0.93-1.08 | <10% |
| Beau, 2014 | 1,501(8) | 2,885(41) | 0.36(OR)  0.17-0.78 | <2 SD@ |
| Cleary, 2014 | 2,996(368) | 3,898(485) | 0.98(OR)  0.85-1.13 | <10% |
| Legge, 2014 | 1,856(138) | 9,437(749) | 0.96(OR)  0.85-1.13 | <10% |
| Nordin, 2014 | 57,554(4,639) | 57,554(4,642) | 1.0(OR)  0.96-1.04 | <10% |
| Ahrens,2014 | 334(30) | 1,285(142) | 0.79(OR)  0.53-1.22 | <10% |
| Trott, 2014 | 6,131(562) | 23,987(2,307) | 0.95(OR)  0.86-1.04 | <10% |
| Adedinsewo,2013 | 916(NA) | 5,422(NA) | 0.83(OR)  0.60-1.17 | <10% |
| Cantu, 2013 | 972(56) | 1,969(125) | 0.9(RR)**  0.6-1.3 | <10% |
| Ludvigsson,2013 | 13,297(1,131) | 7,790(761) | 0.97(OR)  0.9-1.05 | <10% |
| Richards,2013 | 1,125(99) | 1,581(123) | 1.26(OR)  0.94-1.69 | <10% |
| Dodds,2012 | 1,925(124) | 7,722(650) | 0.8(RR)**  0.65-0.97 | <10% |
| Fell, 2012 | 23,265(1937) | 32,068(3,149) | **0.9(RR)****  **0.85-0.96** | <10% |
| Kallen,2012 | 18,612(318) | 136,914(3,066) | 1.04(OR)  0.92-1.17 | <2 SD@ |
| Pasternak_2012  (2)_1# | 330(25) | 330(31) | 0.79(OR)##  0.46-1.37 | <10% |
| Pasternak_2012  (2)_2$ | 6,642(641) | 6,642(657) | 0.97(OR)##  0.87-1.09 | <10% |
| Omer, 2011 | 578(NA). | 3,590(NA) | 0.31(OR)  0.13-0.75 | <10% |

*PY: Person Year

**RR: Risk Ratio

¶ RR: Relative risk

# vaccine administration during the 1^st^ trimester

## POR: Prevalence Odds Ratio

$ vaccine administration during the 2^nd^-3rd trimester

@ Standard Deviation

**S4 (D) Table. Information of studies included in congenital malformation analysis**

| **Name** | **No. vaccinated (event)** | **No. Unvaccinated (event)** | **Effect size,**  **95% CI** | **Administration period**  **(trimester)** |
| --- | --- | --- | --- | --- |
| Steinhoff, 2017 | 1,847(20) | 1,846(18) | 1.11(RR)**  0.59-2.09 | 2nd-3rd |
| Kharbanda, 2017 | 52,856(865) | 373,088(5730) | 1.02(PR)***  0.94-1.10 | 1st |
| Chambers, 2016 | 427(13) | 457(26) | 1.87(RR)**  0.97-3.59 | Whole period |
| Ludvigsson, 2016 | 40,983(2037) | 197,588(2825) | 1.00(OR)  0.95-1.06 | Whole period |
| Fabiani, 2015 | 25942(15) | 1,331,113 (549) | 1.32(OR)  0.78-2.21 | 2nd-3rd |
| Cleary, 2014 | 2,996(66) | 3,898(110) | 0.78(OR)  0.57-1.05 | total |
| Trotta, 2014 | 5,776(260) | 22,145(873) | 1.14(OR)  0.99-1.31 | 2nd-3rd |
| Chambers, 2013 | 338(9) | 188(6) | 0.79(RR)¶  0.26-2.42 | 1^st^ and LMP-DOC## |
| Heikkinen, 2012 | 2,295(56) | 2,213(41) | 1.33(OR)  0.88-2.00 | Whole period |
| Kallen, 2012 | 18,612(108) | 136,914(4768) | 1.01(OR)  0.83-1.23 | 1st |
| Launay, 2012 | 320(4) | 557(3) | 2.34(OR)  0.52-10.51 | 2nd-3rd |
| Lin,2012 | 202(4) | 206(7) | 0.57(OR)  0.17-1.99 | 2nd-3rd |
| Oppermann, 2012 | 321(30) | 1,198(138) | 0.92(OR)  0.58-1.46 | Whole period |
| Pasternak, 2012(2)_1# | 330(18) | 330(15) | 1.21(OR)  0.60-2.45 | 1st |
| Sheffield, 2012 | 8,864(136) | 76,919(1163) | 1.01(OR)  0.85-1.21 | 1st |
| Mackenzie, 2011 | 86(6) | 11(0) | 1.86(OR)  0.10-35.20 | Whole period |
| Munoz, 2005 | 225(0) | 826(15) | 0.12(OR)  0.01-1.95 | 2nd-3rd |
| Deinard, 1981 | 186(19) | 489(67) | 0.72(OR)  0.42-1.23 | NA |
| Louik, 2016 | 2,866(461) | 1,411(250) | 1.01(OR)  0.85-1.21 | 1st |

*vaccine administration during the 1^st^ trimester

**RR: Risk Ratio

***PR: Prevalence Rate

¶ RR: Relative risk

#LMP-DOC: exposure between 1st day of last menstrual period and estimated date of conception

**S4(E) Table. Information of studies included in fetal death analysis**

| **Name** | **No. vaccinated (event)** | **No. Unvaccinated (event)** | **Effect size**  **95% CI** | **Definition of fetal death** |
| --- | --- | --- | --- | --- |
| Steinhoff, 2017 | 1,853(33) | 1,857(31) | 1.07(RR)**  0.66-1.73 | ≥28weeks; stillbirth |
| Madhi, 2014 | 1,044(18) | 1,037(14) | 1.2771(OR)  0.632-2.581 | <28 weeks; miscarriage& stillbirth |
| Heikkinen, 2012 | 2,295(22) | 2,213(15) | 1.44(OR)  0.23–8.90 | stillbirth |
| Getahun, 2019 | 130,996(667) | 116,040(710) | **0.88(OR)**  **0.78-0.99** | Stillbirth |
| Sukumaran, 2018 | 157(50) | 157(58) | 0.96(OR)  0.54-1.69 | Deaths within 6 months age |
| Donahue, 2017 | 485death  (127 vaccinated) | 485 death  (123 unvaccinated) | 0.97(OR)  0.73-1.28 | Spontaneous abortion |
| Ludvigsson, 2015 | 41,183(115) | 234,317(1057) | 0.83(OR)  0.65-1.04 | stillbirth |
| Regan, 2016 | 5,076(3)*** | 52,932(5)*** | **0.49(HR)**  **0.29-0.84** | Stillbirths |
| Baum, 2015 | 34,241(78) | 9,363(25) | 1.05(HR)  0.66-1.65 | ≥22 weeks; stillbirth |
| Fabiani,2015 | 163,928(3)* | 11,733,818 (100)* | 1.45(HR)  0.46-4.62 | ≥22 weeks; stillbirth |
| Beau,2014 | 1,522(13) | 2,890(159) | 0.56(HR)  0.31-1.01 | pregnancy loss |
| Cantu, 2013 | 979(8) | 2,008(15) | 1.09(OR)  0.46-2.59 | ≥20 weeks; stillbirth |
| Fell, 2012 | 23,340(60) | 32,230(139) | **0.66(RR)****  **0.47-0.91** | ≥20 weeks intrauterine death |
| Haberg,2013 | 25,976(78) | 87,335(414) | 0.88(HR)  0.66-1.17 | Fetal death  (miscarriage + stillbirth) |
| Kallen,2012 | 18,612(52) | 136,914(533) | 0.77(OR)  0.57-1.03 | stillbirth |
| Pasternak, 2012(1) | 7,014(7) | 43,633(131) | **0.44 (HR)**  **0.20-0.94** | ≥22 weeks; stillbirth |
| Irving, 2013 | 243(16)  fetal death  (vaccinated) | 243(15)  fetal death  (vaccinated) | 1.23(OR)  0.53-2.89 | spontaneous abortion |
| Chambers,2013 | 831(1) | 191(1) | 0.23(RR)¶,  0.01-3.93 | stillbirth |
| Deinard,1981 | 189(1) | 517(1) | 2.74(OR)  0.17-44.10 | stillbirth |

*PD: Pregnant days at risk

**RR: Risk Ratio, ¶,RR: Relative Risk

***Stillbirths per 100,000 pregnancy-days
