# Supplementary material for: Effects of maternal influenza vaccination on adverse birth outcomes: A systematic review and Bayesian meta-analysis
Source: PLoS One. 2019 Aug 14;14(8):e0220910. doi: 10.1371/journal.pone.0220910 (PMC6693758; doi:10.1371/journal.pone.0220910)
Supplement: S5 Table — (DOCX) [file pone.0220910.s005.docx]

S5 Table. Meta-regression results of gestational period when vaccination given on congenital malformation

| Birth outcome | Study | Bayesian random-effect meta-regression model (Odds ratio) | | | | |  |
| --- | --- | --- | --- | --- | --- | --- | --- |
|  |  | Posterior median | | 95%  Credible interval | | | P(OR<1) |
| Congenital  malformation | SD( τ ) | 0.1187 | | | | | |
|  | Overall | 0.865 | 0.192 | | 2.757 | 0.618 | |
|  | RCT overall | 0.905 | 0.207 | | 2.550 | 0.587 | |
|  | Cohort overall | 0.841 | 0.212 | | 2.496 | 0.641 | |
|  | Case-control overall | 0.849 | 0.195 | | 2.703 | 0.630 | |

 SD( τ ) : Standard deviation between studies (estimation of heterogeneity between studies)
