# Supplementary material for: Effects of maternal influenza vaccination on adverse birth outcomes: A systematic review and Bayesian meta-analysis
Source: PLoS One. 2019 Aug 14;14(8):e0220910. doi: 10.1371/journal.pone.0220910 (PMC6693758; doi:10.1371/journal.pone.0220910)
Supplement: S6 Table — (DOCX) [file pone.0220910.s006.docx]

S6(A) Table. Summary estimate of conventional meta-analysis with random effects model constituted by cohort studies (PTB)

| Cohort studies | Odds ratio | | | p-value | I^2^ |
| --- | --- | --- | --- | --- | --- |
|  | OR | 95% Confidence interval | |  |  |
| Overall summary estimates | 0.91 | 0.85 | 0.98 | 0.015 | 89.806 |
| Getahun, 2019 | 1.02 | 0.99 | 1.05 |  |  |
| McHugh, 2019 | 1.10 | 0.92 | 1.31 |  |  |
| Arriola, 2017 | 0.88 | 0.73 | 1.06 |  |  |
| Zerbo, 2017 | 0.69 | 0.66 | 0.72 |  |  |
| McHugh, 2017 | 1.14 | 0.12 | 1.27 |  |  |
| Chambers, 2016 | 1.23 | 0.75 | 2.02 |  |  |
| Vazquez-Benitez, 2016 | 0.91 | 0.83 | 1.00 |  |  |
| Olsen, 2016 | 0.70 | 0.57 | 0.87 |  |  |
| Baum, 2015 | 1.00 | 0.89 | 1.12 |  |  |
| Fabiani,2015 | 1.15 | 0.95 | 1.39 |  |  |
| Ma, 2014 | 0.28 | 0.01 | 6.99 |  |  |
| Ahrens, 2014 | 1.03 | 0.83 | 2.05 |  |  |
| Beau,2014 | 0.82 | 0.64 | 1.06 |  |  |
| Cleary, 2014 | 0.71 | 0.58 | 0.88 |  |  |
| Legge, 2014 | 0.75 | 0.60 | 0.94 |  |  |
| Nordin, 2014 | 0.97 | 0.93 | 1.02 |  |  |
| Adedinsewo,2013 | 0.83 | 0.60 | 1.17 |  |  |
| Cantu, 2013 | 1.20 | 0.90 | 1.60 |  |  |
| Ludvigsson,2013 | 0.99 | 0.89 | 1.10 |  |  |
| Chambers, 2013 | 3.28 | 1.25 | 8.63 |  |  |
| Heikkinen, 2012 | 0.75 | 0.55 | 0.99 |  |  |
| Oppermann, 2012 | 0.98 | 0.64 | 1.49 |  |  |
| Dodds,2012 | 0.84 | 0.69 | 1.02 |  |  |
| Fell, 2012 | 0.95 | 0.88 | 1.02 |  |  |
| Kallen,2012 | 0.86 | 0.77 | 0.96 |  |  |
| Lin,2012 | 0.54 | 0.27 | 1.07 |  |  |
| Pasternak, 2012_(2)_1 | 1.00 | 0.84 | 1.19 |  |  |
| Pasternak, 2012_(2)_2 | 0.97 | 0.87 | 1.09 |  |  |
| Omer, 2011 | 0.83 | 0.55 | 1.26 |  |  |

S6(B) Table. Summary estimate of conventional meta-analysis with random effects model constituted by cohort studies (LBW)

| Cohort studies | Odds ratio | | | p-value | I^2^ |
| --- | --- | --- | --- | --- | --- |
|  | OR | 95% Confidence interval | |  |  |
| Overall summary estimates | 0.93 | 0.87 | 1 | 0.039 | 34.336 |
| McHugh, 2019 | 1.05 | 0.76 | 1.05 |  |  |
| Arriola, 2017 | 0.83 | 0.62 | 1.06 |  |  |
| McHugh, 2017 | 1.04 | 0.85 | 1.26 |  |  |
| Zerbo, 2017 | 0.95 | 0.86 | 1.06 |  |  |
| Baum, 2015 | 1.05 | 0.90 | 1.21 |  |  |
| Fabiani, 2015 | 0.92 | 0.69 | 1.23 |  |  |
| Ma, 2014 | 1.72 | 0.15 | 19.21 |  |  |
| Legge, 2014 | 0.73 | 0.56 | 0.95 |  |  |
| Cantu, 2013 | 1.00 | 0.80 | 1.50 |  |  |
| Ludvigsson, 2013 | 0.91 | 0.79 | 1.04 |  |  |
| Heikkinen, 2012 | 0.88 | 0.61 | 1.26 |  |  |
| Lin, 2012 | 0.55 | 0.29 | 1.05 |  |  |
| Dodds, 2012 | 0.74 | 0.58 | 0.95 |  |  |
| Kallen, 2012 | 0.86 | 0.77 | 0.96 |  |  |
| Pasternak, 2012_(2)_1 | 0.83 | 0.41 | 1.67 |  |  |
| Pasternak, 2012_(2)_2 | 1.14 | 0.94 | 1.38 |  |  |

S6(C) Table. Summary estimate of conventional meta-analysis with random effects model constituted by cohort studies (SGA)

| Cohort studies | Odds ratio | | | p-value | I^2^ |
| --- | --- | --- | --- | --- | --- |
|  | OR | 95% Confidence interval | |  |  |
| Overall summary estimates | 0.97 | 0.94 | 1.01 | 0.102 | 43.632 |
| Getahun, 2019 | 0.99 | 0.96 | 1.02 |  |  |
| McHugh,2019 | 0.99 | 0.86 | 1.15 |  |  |
| Arriola, 2017 | 1.02 | 0.83 | 1.24 |  |  |
| Zerbo, 2017 | 0.98 | 0.92 | 1.04 |  |  |
| Chambers, 2016 | 1.49 | 0.93 | 2.39 |  |  |
| Olsen, 2016 | 1.25 | 0.91 | 1.72 |  |  |
| Vazquez-Benitez, 2016 | 0.97 | 0.91 | 1.03 |  |  |
| Baum, 2015 | 1.17 | 0.98 | 1.40 |  |  |
| Beau, 2014 | 0.36 | 0.17 | 0.78 |  |  |
| Cleary, 2014 | 0.98 | 0.85 | 1.13 |  |  |
| Legge, 2014 | 0.96 | 0.79 | 1.16 |  |  |
| Nordin, 2014 | 1.00 | 0.96 | 1.04 |  |  |
| Ahrens,2014 | 0.79 | 0.53 | 1.20 |  |  |
| Trott, 2014 | 0.95 | 0.86 | 1.04 |  |  |
| Adedinsewo.2013 | 0.83 | 0.60 | 1.17 |  |  |
| Cantu, 2013 | 0.90 | 0.60 | 1.30 |  |  |
| Ludvigsson,2013 | 0.97 | 0.90 | 1.05 |  |  |
| Richards,2013 | 1.14 | 0.87 | 1.51 |  |  |
| Dodds,2012 | 0.80 | 0.65 | 0.97 |  |  |
| Fell, 2012 | 0.90 | 0.85 | 0.96 |  |  |
| Kallen,2012 | 1.04 | 0.92 | 1.17 |  |  |
| Pasternak_2012(2)_1 | 0.79 | 0.46 | 1.37 |  |  |
| Pasternak_2012(2)_2 | 0.97 | 0.87 | 1.09 |  |  |
| Omer, 2011 | 0.31 | 0.13 | 0.75 |  |  |

S6 (D) Table. Summary estimate of conventional meta-analysis with random effects model constituted by cohort studies (Congenital malformation)

| Cohort studies | Odds ratio | | | p-value | I^2^ |
| --- | --- | --- | --- | --- | --- |
|  | OR | 95% Confidence interval | |  |  |
| Overall summary estimates | 1.02 | 0.97 | 1.09 | 0.402 | 14.586 |
| Heikkinen, 2012 | 1.33 | 0.88 | 2.00 |  |  |
| Ludvigsson, 2016 | 1.00 | 0.95 | 1.06 |  |  |
| Oppermann, 2012 | 0.92 | 0.58 | 1.46 |  |  |
| Chambers, 2016 | 1.87 | 0.97 | 3.59 |  |  |
| Kharbanda, 2017 | 1.02 | 0.94 | 1.10 |  |  |
| Fabiani, 2015 | 1.32 | 0.78 | 2.21 |  |  |
| Munoz, 2005 | 0.12 | 0.01 | 1.95 |  |  |
| Cleary, 2014 | 0.78 | 0.57 | 1.05 |  |  |
| Kallen, 2012 | 1.01 | 0.83 | 1.23 |  |  |
| Pasternak, 2012(2)_1 | 1.21 | 0.60 | 2.45 |  |  |
| Deinard, 1981 | 0.72 | 0.42 | 1.23 |  |  |
| Mackenzie, 2011 | 1.86 | 0.10 | 35.20 |  |  |
| Launay, 2012 | 2.34 | 0.52 | 10.51 |  |  |
| Sheffield, 2012 | 1.01 | 0.85 | 1.21 |  |  |
| Trotta, 2014 | 1.14 | 0.99 | 1.31 |  |  |
| Chambers, 2013 | 0.79 | 0.26 | 2.42 |  |  |
| Lin, 2012 | 0.57 | 0.17 | 1.99 |  |  |

S6 (E) Table. Summary estimate of conventional meta-analysis with random effects model constituted by cohort studies (Fetal death)

| Cohort studies | Odds ratio | | | p-value | I^2^ |
| --- | --- | --- | --- | --- | --- |
|  | OR | 95% Confidence interval | |  |  |
| Overall summary estimates | 0.81 | 0.72 | 0.90 | <0.001 | 15.164 |
| Getahun,2019 | 0.88 | 0.78 | 0.99 |  |  |
| Regan, 2016_Clin Inf Dis | 0.49 | 0.29 | 0.84 |  |  |
| Baum, 2015 | 1.05 | 0.66 | 1.65 |  |  |
| Fabiani,2015 | 1.45 | 0.46 | 4.62 |  |  |
| Ludvigsson, 2015 | 0.83 | 0.65 | 1.04 |  |  |
| Beau,2014 | 0.56 | 0.31 | 1.01 |  |  |
| Cantu, 2013 | 1.09 | 0.46 | 2.59 |  |  |
| Chambers,2013 | 0.23 | 0.01 | 3.93 |  |  |
| Haberg,2013 | 0.88 | 0.66 | 1.17 |  |  |
| Fell, 2012 | 0.66 | 0.47 | 0.91 |  |  |
| Heikkinen, 2012 | 1.44 | 0.23 | 8.90 |  |  |
| Kallen,2012 | 0.77 | 0.57 | 1.03 |  |  |
| Pasternak, 2012(1) | 0.44 | 0.2 | 0.94 |  |  |
| Deinard,1981 | 2.74 | 0.17 | 44.10 |  |  |
