# Supplementary figures and images for: Effects of maternal influenza vaccination on adverse birth outcomes: A systematic review and Bayesian meta-analysis
Source: PLoS One. 2019 Aug 14;14(8):e0220910. doi: 10.1371/journal.pone.0220910 (PMC6693758; doi:10.1371/journal.pone.0220910)

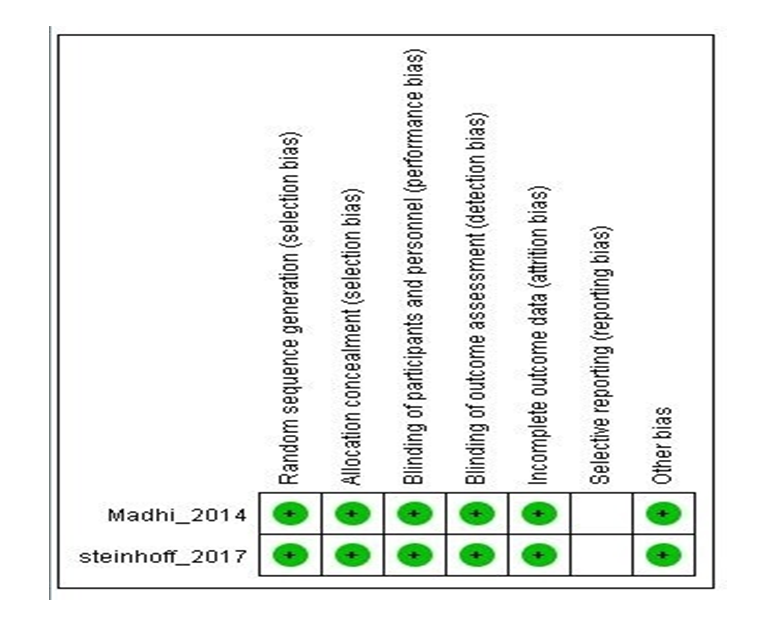

Supplement: S1 Fig — (TIF) [file pone.0220910.s007.tif]

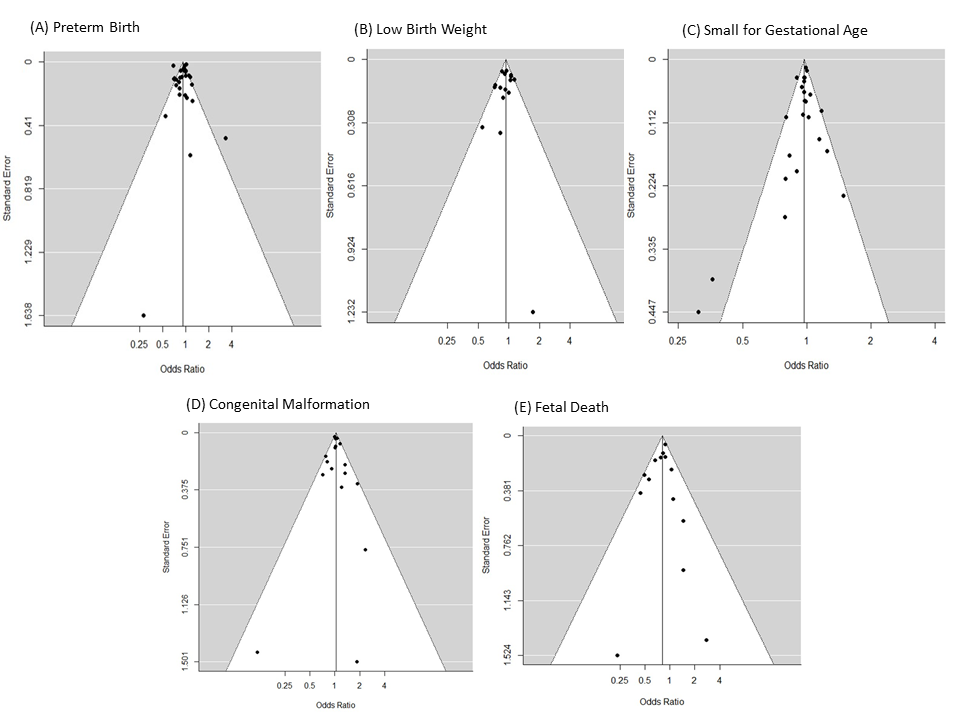

Supplement: S2 Fig — (TIF) [file pone.0220910.s008.tif]
